# Omega-3 Docosahexaenoic Acid (DHA) Impedes Silica-Induced Macrophage Corpse Accumulation by Attenuating Cell Death and Potentiating Efferocytosis

**DOI:** 10.3389/fimmu.2020.02179

**Published:** 2020-10-06

**Authors:** Lichchavi D. Rajasinghe, Preeti S. Chauhan, Kathryn A. Wierenga, Augustus O. Evered, Shamya N. Harris, Melissa A. Bates, Mikhail A. Gavrilin, James J. Pestka

**Affiliations:** ^1^Department of Food Science and Human Nutrition, Michigan State University, East Lansing, MI, United States; ^2^Institute for Integrative Toxicology, Michigan State University, East Lansing, MI, United States; ^3^Department of Biochemistry and Molecular Biology, Michigan State University, East Lansing, MI, United States; ^4^Division of Pulmonary, Critical Care and Sleep Medicine, Ohio State University, Columbus, OH, United States; ^5^Department of Microbiology and Molecular Genetics, Michigan State University, East Lansing, MI, United States

**Keywords:** alveolar macrophage, autoimmunity, phagocytosis, silica, inflammation, apoptosis, pyroptosis, necrosis

## Abstract

Airway exposure of lupus-prone NZBWF1 mice to crystalline silica (cSiO_2_), a known trigger of human autoimmune disease, elicits sterile inflammation and alveolar macrophage death in the lung that, in turn, induces early autoimmune onset and accelerates lupus progression to fatal glomerulonephritis. Dietary supplementation with docosahexaenoic acid (DHA), a marine ω-3 polyunsaturated fatty acid (PUFA), markedly ameliorates cSiO_2_-triggered pulmonary, systemic, and renal manifestations of lupus. Here, we tested the hypothesis that DHA influences both cSiO_2_-induced death and efferocytotic clearance of resultant cell corpses using three murine macrophage models: (i) primary alveolar macrophages (AM) isolated from NZBWF1 mice; (ii) self-renewing AM-like Max Planck Institute (MPI) cells isolated from fetuses of C57BL/6 mice, and (iii) RAW 264.7 murine macrophages, a virus-transformed cell line derived from BALB/c mice stably transfected with the inflammasome adaptor protein ASC (RAW-ASC). Incubation with cSiO_2_ at 25 and 50 μg/ml for 6 h was found to dose-dependently induce cell death (*p* < 0.05) in all three models as determined by both acridine orange/propidium iodide staining and release of lactate dehydrogenase into cell culture supernatant. Pre-incubation with DHA at a physiologically relevant concentration (25 μM) significantly reduced cSiO_2_-induced death (*p* < 0.05) in all three models. Cell death induction by cSiO_2_ alone and its suppression by DHA were primarily associated with caspase-3/7 activation, suggestive of apoptosis, in AM, MPI, and RAW-ASC cells. Fluorescence microscopy revealed that all three macrophage models were similarly capable of efferocytosing RAW-ASC target cell corpses. Furthermore, MPI effector cells could likewise engulf RAW-ASC target cell corpses elicited by treatment with staurosporine (apoptosis), LPS, and nigericin (pyroptosis), or cSiO_2_. Pre-incubation of RAW-ASC target cells with 25 μM DHA prior to death induced by these agents significantly enhanced their efferocytosis (*p* < 0.05) by MPI effector cells. In contrast, pre-incubating MPI effector cells with DHA did not affect engulfment of RAW-ASC target cells pre-incubated with vehicle. Taken together, these findings indicate that DHA at a physiologically relevant concentration was capable of attenuating macrophage death and could potentiate efferocytosis, with the net effect of reducing accumulation of cell corpses capable of eliciting autoimmunity.

## Introduction

Systemic lupus erythematosus (lupus) is an autoimmune disease characterized by the loss of immunological tolerance and generation of a diverse pathogenic autoantibody repertoire. This disease often manifests as alternating episodes of remission and flaring, eventually causing irreversible damage to multiple organs including the kidney, skin, heart, and brain. Lupus pathogenesis has been linked to both excessive cell death and defective removal of dying/dead cells via a process known as efferocytosis (1–7). Faulty clearance of cell corpses results in secondary necrosis coupled with unmasking of intracellular antigens that, in the context of chronic inflammation, is capable of driving adaptive immune responses yielding autoantibodies; these bind to self-antigens and form immune complexes that are injurious to tissues (8, 9).

Development of lupus depends on genetic pre-disposition, but the initiation and progression of autoimmunity can be positively or negatively influenced by environmental factors (10). One environmental factor that has been epidemiologically associated with lupus and other autoimmune diseases is crystalline silica (cSiO_2_), a respirable particle frequently inhaled by workers in occupations such as mining, construction, and farming (11, 12). Exposure to respirable cSiO_2_ induces pulmonary toxicity in mice associated with unresolved inflammation and cell death (13–16). Previous studies in lupus-prone mice have established that inhalation of this particle rapidly accelerates the onset and progression of autoimmunity (17–19). More recently, we have demonstrated that intranasal cSiO_2_ instillation of female NZBWF1 mice induces extensive pulmonary inflammation that triggers autoimmunity and glomerulonephritis 3 months earlier than vehicle-instilled controls (20, 21). The pulmonary response is associated sequentially with robust proinflammatory cytokine, chemokine, and interferon-related gene expression, ectopic lymphoid tissue development, and diverse autoantibody production, driving systemic autoimmune disease progression and culminating in end-stage glomerulonephritis (20, 22, 23).

Tissue-resident alveolar macrophages (AMs) play a primary defensive role against inhaled foreign particles, including cSiO_2_, in maintaining immunological homoeostasis and host defense in the lungs (24). cSiO_2_ exposure induces AM death by apoptosis, pyroptosis, and necrosis (16, 25, 26). In addition, AM play essential roles in efferocytosis of dead and dying cells from the lung to prevent untoward inflammatory and immune responses (27). By clearing the cell corpses, AMs limit inflammation and prevent secondary necrosis of apoptotic cells (28, 29). Accordingly, increased cell death and/or inefficient efferocytosis might contribute to cSiO_2_-triggered autoimmunity.

Diet is another environmental factor that can modulate autoimmunity. Dietary supplementation with the omega-3 (ω-3) polyunsaturated fatty acid (PUFA), docosahexaenoic acid (DHA), markedly attenuates cSiO_2_-triggered pulmonary, systemic, and renal manifestations of lupus (20, 22, 30, 31). To better understand how DHA suppresses cSiO_2_-triggered lupus, it is essential to discern how this ω-3 PUFA influences the inflammation and cell death induced by cSiO_2_ in the lung. ω-3s and their metabolites have been widely reported to enhance the ability of AMs to be recognized as dying cells and be removed via efferocytosis (32–34).

Here, we tested the hypothesis that DHA influences both cSiO_2_-induced death and efferocytotic clearance of resultant cellular corpses. Three distinct macrophage models were employed in this study. For *in vitro* or *ex vivo* efferocytosis and cell death studies, AM are an appropriate model because they represent the phenotype of macrophages in the lung alveoli (35) and their responses in culture correlate with disease pathogenesis *in vivo* (36). However, AM recoveries are typically <10^6^ cells per mouse, making it difficult to obtain sufficient quantities for the mechanistic studies of cell death and efferocytosis such as those performed here. Therefore, two other macrophage models were employed as AM surrogates. During murine development, long-lived AMs originate from fetal yolk-sac precursors that migrate from the liver to the lung shortly after birth. Self-renewing AM-like Max Planck Institute (MPI) cells, developed by isolating fetal monocytes and culturing for 2 weeks in GM-CSF, express surface markers and gene expression seen in AMs (37, 38). The RAW 264.7 murine clone has been used as a model for macrophages in more than 10,000 publications since it was established in 1977 (39). In a prior study (23), we transfected RAW 264.7 cells with the gene encoding the protein ASC, rendering them capable of mounting an inflammasome response similar to that of primary AMs (40, 41). The resultant *in vitro* findings presented here indicate that DHA's ameliorative effects on cSiO_2_-induced lupus might be linked to its capacity to reduce autoantigenic cell corpse accumulation in the lung by both attenuating macrophage death and potentiating efferocytosis.

## Materials and Methods

### cSiO_2_

cSiO_2_ (Min-U-Sil-5, Pennsylvania Glass Sand Corp, Pittsburgh, PA) was formulated using a previously described protocol (42). Briefly, it was suspended in 1M HCl and heated to 100°C for 1 h. After cooling, the particles were washed three times with autoclaved water, dried overnight at 200°C, and suspended in sterile Dulbecco's phosphate-buffered saline (DPBS, Thermo Fisher Scientific, Waltham, MA). For addition to cultures, the suspensions were thoroughly vortexed, sonicated for 1 min, and added dropwise to wells to attain required concentrations.

### Preparation of DHA-BSA Complexes

DHA-bovine serum albumin (BSA) complexes (3:1) were formulated as described previously (43, 44). Fatty acid-free, endotoxin-free BSA (Millipore Sigma, Burlington, MA) was dissolved in Roswell Park Memorial Institute (RPMI) 1640 medium (Thermo Fisher Scientific, Waltham, MA) at 15% (w/v). DHA (Cayman Chemical, Ann Arbor, MI) was dissolved in EtOH at 11.76 mg/ml. Stock solution corresponding to 20 mg DHA was transferred to a glass test tube and dried under N_2_ gas. DHA was dissolved in 4 ml of 0.05 M Na_2_CO_3_ to yield concentration of 5 mg/ml. The solution was flushed with N_2_ gas, vortexed, and incubated for 1 h at room temperature. DHA in Na_2_CO_3_ and 15% BSA in RPMI were combined in serum-free RPMI to achieve final concentrations of 2.5 mM DHA and 0.833 mM BSA (3:1 molar ratio). After flushing with N_2_ and gently mixing for 30 min, the DHA-BSA complex solution was filter sterilized, aliquoted, and stored under N_2_ at −20°C for ≤3 months.

### Animals

All experimental protocols involving NZBWF1 and C57BL/6 mice were reviewed and approved by the Institutional Animal Care and Use Committee at Michigan State University in accordance with the National Institutes of Health guidelines (AUF # PROTO201800113). Six-week-old female NZBWF1 mice and C57BL/6 mice were purchased from Jackson Laboratories (Bar Harbor, ME). Upon arrival, mice were sheltered four per cage with *ad libitum* food and water. Animal facilities were maintained at continuous temperature (21–24°C) and humidity (40–55%) with a 12 h light/dark cycle.

### Cell Cultures

#### Alveolar Macrophages

AMs were isolated from bronchoalveolar lavage fluid (BALF) of female NZBWF1 mice as described by Zhang et al. (45). BALF was pelleted by centrifugation at 500 × g at 4°C for 10 min, suspended, and cultured overnight at 37°C/humidified air in 5% CO_2_ in RPMI medium containing 1% penicillin-streptomycin (Pen-Strep, Thermo Fisher Scientific), 30 ng/ml of granulocyte-macrophage colony-stimulating factor (GM-CSF, R&D Systems, Minneapolis, MN), and 10% fetal bovine serum (FBS, R&D Systems). Complete RPMI Media (RPMI, 10% FBS, 1% Pen-Strep) with 30 ng/ml of GM-CSF was refreshed every 2–3 days, discarding non-adherent cells. When the proliferating adherent monolayer reached 80–90% confluence (every 4–6 days), cells were sub-cultured. Cells were passaged no more than five times.

#### Max Planck Institute Cells

AM-like MPI cells were obtained as described by Fejer et al. (37). Briefly, C57BL/6 fetal livers were excised at 14–18 gestational days and dissociated into a single cell suspension in ice-cold, sterile DPBS. Cells were passed through a 40 μm filter, washed twice with sterile DBPS, and plated in 100 mm plates (one plate per liver). Complete RPMI was supplemented with 30 ng/ml GM-CSF to induce differentiation of fetal liver monocytes into MPI cells. Complete RPMI with GM-CSF was refreshed every 2–3 days, discarding non-adherent cells. When the proliferating adherent monolayer reached 80–90% confluence (every 4–6 days), cells were sub-cultured. After ~2 weeks, the adherent cells developed a distinct “fried-egg” morphology akin to AM and expressed common surface markers of AM phenotype namely, CD11c and Siglec F. At this point, MPI cells could be continually sub-cultured for use in assays or cryopreserved. MPI cells from passage 10 to 20 were used for this study.

#### RAW-ASC Cells

Murine-derived wild-type RAW 264.7 (RAW-WT) (American Type Culture Collection; ATCC® TIB-71™) were stably transfected with a fusion C-terminus of cyan fluorescent protein (CFP)-ASC protein to generate RAW-ASC cells. Briefly, the open-reading frame of ASC was amplified from cDNA by polymerase chain reaction (PCR) and inserted at the CFP of pLenti-CFP plasmid generated on the basis of pLenti6/V5 (Invitrogen Life Technologies, Carlsbad, CA) resulting in a fusion CFP-ASC protein, as previously reported (46, 47). Cell types were cultured in complete RPMI medium and sub-cultured every 2–4 days.

#### DHA Treatment

AMs, MPI, or RAW-ASC macrophages were seeded at 1.5 × 10^5^ cells/well in 24-well plate, or 1.7 × 10^4^ cells/well in 96-well plate to achieve 70–90% confluency at the time of treatment. AMs and MPI cells were grown in complete RPMI or serum-reduced RPMI (phenol red-free RPMI 1640, 0.25% FBS, 1% penicillin-streptomycin) media supplemented with GM-CSF and RAW-ASC macrophages were grown in complete or serum-reduced RPMI media. After 24 h in complete RPMI or complete RPMI with GM-CSF, wells were washed, and media was switched to fatty acid-supplemented serum-deprived RPMI supplemented with or without GM-CSF. DHA-BSA complex (3:1 molar ratio) equivalent to 25 μM or vehicle control (Veh) with the corresponding concentration of BSA was added to serum-deprived media with or without GM-CSF (23). After 24 h in fatty acid-supplemented serum-deprived RPMI media, cells were washed once with sterile DPBS and subjected to treatments and analyses.

### Cell Death Studies

#### Cell Viability Determination With Propidium Iodide (PI)/Acridine Orange (AO)

PI is a commonly used red fluorescent stain to examine cell death by binding DNA of dead/dying cells with increased membrane permeability. AO is a green fluorescent stain that penetrates intact membranes to visualize live cells. AM, MPI, RAW-ASC cells were incubated with 25 μM DHA or Veh, followed by exposure to 0, 25, or 50 μg/ml cSiO_2_ for 6 h in 24-well plates. Half of the cell media (500 μl) was then removed from each well and replaced with an equal volume of 0.5μg/ml PI (Immunochemistry Technologies, LLC, Bloomington, MN) and 1 μM AO (Thermo Fisher) suspended in serum-deprived RPMI media, resulting in 0.25 μg/ml of PI and 0.5 μM of AO. The plate was spun at 100 × g for 2 min to ensure cell adherence before imaging. The dead (red, PI, Ex: 585/29 Em: 628/32) and live (green, AO, Ex: 470/22 Em: 525/50) cells were visualized using the EVOS FL Auto 2 Cell Imaging System (Thermo Fisher Scientific), keeping the cells at optimal growing conditions in the incubation space while taking images (5% CO_2_, 37°C, >80% humidity). Images were captured using the 20× lens objective, taking nine images per well to ensure accurate depictions of the whole well. These images were analyzed via Celleste Image Analysis software (Thermo Fisher), where the total number of dead and live cells for each treatment group per cell line were counted and the cell death % was calculated.

#### Lactate Dehydrogenase (LDH) Assay

LDH release measurements were also employed to assess effects of DHA on cSiO_2_-induced death as previously described (23). Briefly, AM, MPI, RAW-ASC cells were incubated with 25 μM DHA or Veh, followed by exposure to 0, 25, or 50 μg/ml cSiO_2_ for 6 h in 24-well plates. Additionally, 20 μl of 10% Triton-X (Millipore Sigma) was added to some wells, designated as maximum kill (MK). Media were collected from sample and MK wells and 50 μl transferred to untreated, flat-bottomed 96-well plate. Serum-deprived RPMI was used as a sample blank and serum-deprived RPMI with 10% Triton-X was used as the MK blank. 100 μl of LDH substrate (2 mM iodonitrotetrazolium chloride, 3.2 mM β-nicotinamide adenine dinucleotide sodium salt, 160 mM lithium lactate, 15 μM 1-methoxyphenazine methosulfate in 0.2 M Tris-HCl, pH 8.2) was added to each well. Plates were then measured using a FilterMax F3 Multimode plate reader (Molecular Devices, San Jose, CA) at an absorbance wavelength of 492 nm. Cytotoxicity was determined as follows: 100% ^*^ [(sample_abs_-sample blank_abs_)/(MK_abs_-MK blank_abs_)].

#### MTS Assay

Cell viability was assessed using a CellTiter 96® AQueous One Solution Cell Proliferation Assay (Promega, Madison, WI). CellTiter 96® AQueous One Solution Reagent contains the tetrazolium compound MTS which is reduced by viable cells to a colored formazan product that is soluble in the tissue culture medium. Briefly, RAW-ASC cells were incubated with 25 μM DHA or Veh, followed by exposure to 50 μg/ml cSiO_2_ for 16 h, 0.5 μM staurosporine (R&D Systems) for 8 h and 5.0 μM nigericin (Millipore Sigma) for 6 h following priming with 20 ng/ml LPS (from *Salmonella enterica* serotype typhimurium containing <1% protein impurities, Millipore Sigma) for 2 h in 24-well plates. Immediately following above treatments, the plate was centrifuged at 220 × g for 3 min and half of the cell media (600 μl) removed from each well. Then, 20 μl of One Solution Reagent was added directly to the culture plate. After 30–60 min incubation at 37°C, the absorbance was read on an EnSpire™ Multilabel Plate Reader (PerkinElmer Inc., Waltham, MA) at an absorbance wavelength of 490 nm. The quantity of formazan product, as measured by the absorbance at 490 nm, is directly proportional to the number of living cells in culture. Cell viability was calculated as a percentage of untreated control cells.

#### Caspase-3/7 Activation

Caspase-3/7 activity was determined in cSiO_2_-treated AM, MPI, and RAW-ASC cells using the Caspase-Glo® 3/7 assay (Promega). Briefly, AM, MPI, and RAW-ASC cells were incubated with 25 μM DHA or Veh, followed by exposure to 0, 25, or 50 μg/ml cSiO_2_ for 6 h in a white-walled 96-well plate for Caspase-3/7 assay with 0.5 μM staurosporine for 6 h used as a positive control. Serum-deprived RPMI was used as a sample blank. To determine caspase-3/7 activation, half of the culture medium (50 μl) was removed and 50 μl of Caspase-Glo® 3/7 Reagent was added to each well of a white-walled 96-well plate containing 100 μl of blank. Luminescence was read after 1 h using an EnSpire™ Multilabel Plate Reader. Caspase-3/7 activity was reported after subtracting the blank values.

#### Caspase-1 Activation

Caspase-1 activity was determined in cSiO_2_ treated unprimed and LPS-primed MPI and RAW-ASC cells using Caspase-Glo® 1 luminescence assay kit (Promega). In brief, MPI and RAW-ASC cells were incubated with 25 μM DHA or Veh, followed by exposure to 0, 25, or 50 μg/ml cSiO_2_ for 6 h following priming with 20 ng/ml LPS or Veh for 2 h in a white-walled 96-well plate. Nigericin (10 ng/ml) treatment for 1 h following priming was used as the positive control. Serum-deprived RPMI was used as a sample blank. To assess caspase-1 activation, half of the culture medium (50 μl) was removed and 50 μl of Caspase-Glo® 1 Reagent + YVAD-CHO mixture was added to each well. Luminescence was read after 1 h as described above and caspase-1 activity reported after subtracting the blank values.

#### IL-1β Release

MPI and RAW-ASC cells were incubated with 25 μM DHA or Veh, followed by priming with 20 ng/ml LPS for 2 h and exposure to 0, 25, or 50 μg/ml cSiO_2_ for 6 h. Treatment with nigericin (10 μM) for 1 h following LPS priming was used as the positive control. After incubation, cell supernatant was collected and IL-1β release was measured for MPI and RAW-ASC cells using the mouse IL-1β/IL-1F2 DuoSet® ELISA (R&D Systems) per manufacturer's instructions.

### Efferocytosis Measurement by Fluorescence Microscopy

#### Labeling of RAW ASC Target Cells

RAW-ASC cells were gathered from 100 mm cell culture dishes by scraping and rinsing with DPBS into a 50 ml conical centrifuge tube. The cells were then centrifuged for 8 min at 220 × g and resuspended in 10 ml of IncuCyte pHrodo Wash Buffer (Essen Bioscience, Ann Arbor, MI) and then centrifuged for another 8 min. Cells were then resuspended in IncuCyte pHrodo Labeling Buffer (Essen Bioscience) at a cell density of 1 × 10^6^ cells/ml. IncuCyte pHrodo Red succinimidyl ester (pHrodo Red SE, Essen Bioscience) was added to cell suspension in the Labeling Buffer at 0.5 μl/ml and incubated for 1 h at 37°C in the dark while inverting every 15 min. After incubating, the cells were brought up to 5× the labeling buffer volume with complete RPMI media and centrifuged for 8 min at 220 × g to wash away unbound dye.

Cells were then cultured overnight in 100 mm culture plates at a concentration of 3.2 × 10^5^ cells/ml. The next day, the target cells were incubated for 24 h with DHA or Veh in serum-deprived medium as described above. Death was then induced in target cells by treatment with (i) 50 μg/ml cSiO_2_ for 16 h to achieve extensive death, (ii) 0.5 μM staurosporine for 8 h to elicit apoptotic cell corpses or with (iii) 20 ng/ml LPS for 2 h followed by 10 μM nigericin for 6 h to generate pyroptotic cell corpses. After cell death had been induced, target cells were collected by centrifugation and resuspended in 2 ml of serum reduced media. Total protein from each sample (DHA- or Veh-treated cells) was measured using a Pierce™ BCA Protein Assay Kit (Thermo Fisher Scientific) to normalize the cell density. For time course studies, cells were exposed directly to the cell death inducer without DHA pre-incubation and necrotic corpses were generated by heating cells at 90°C for 3 min using a block incubator (Thermo Fisher Scientific). Cell viability and mechanisms of death were assessed and confirmed using microscopic observation (morphology and AO/PI staining), cell viability assessment with MTS, caspase-1, and caspase-3/7 as described above (see Supplementary Figures 4–6).

#### Effector Cell Labeling

AM, MPI, and RAW-ASC macrophages were compared initially as effector cells (i.e., viable phagocytes) and then MPI cells were used for subsequent efferocytosis experiments. Macrophages were gathered from 80% confluent 10 mm plates by scraping and rinsing with PBS into a 50 ml conical centrifuge tube and centrifuged for 8 min at 220 × g. The cells were then washed with 10 ml of PBS and centrifuged again to remove trace amounts of FBS from their initial media suspension. The cells were then suspended in complete RPMI media 10 μM and 1% Pen-Strep and the cell density was adjusted to 1 × 10^6^ cells/ml. 1 μL of 1 mM CFSE green stock solution (Invitrogen Life Technologies, Carlsbad, CA) was added per ml of the cell suspension to yield a final concentration of 1 μM. The cells were then incubated in the dark for 30 min at 37°C; the tube was agitated every 10 min to ensure even labeling throughout the media. After incubation, the cells were washed with complete RPMI. The cells were then centrifuged for 8 min and resuspended in complete RPMI media with (AM and MPI) or without GM-CSF (RAW-ASC). The cell density was adjusted to 8 × 10^4^ cells/ml and plated onto a black, clear-bottom, 24-well plate to adhere overnight. The next day, effector cells were examined under the EVOS FL Auto 2 Cell Imaging System using the GFP light cube (Ex: 470/22 Em: 525/50) to confirm the labeling process and incubated for 24 h with DHA or Veh serum-deprived medium as described above.

#### Efferocytosis Assessment by Fluorescent Microscopy

2.4 × 10^5^ target cell corpses were added to the effector cells at 1:4 ratio (effector: target) and then centrifuged for 1 min at 100 × g and efferocytosis measured over time. For the time course studies, efferocytosis was examined at 0, 30, 60, and 120 min after co-culturing. For the DHA pre-treatment experiments, efferocytosis was examined at 1h following co-culturing of target and effector cells pre-incubated with either Veh or DHA. Following the co-culturing, media was removed, the well-washed twice with PBS, and 1 ml Fluoro-Brite media (Thermo Fisher Scientific) was added to each well. The engulfed target cells (bright red, pHrodo red dye) and effector cells (green, CFSE green) were visualized using GFP light cube (Ex: 470/22 nm Em: 525/50 nm) and Texas red light cube (Ex: 585/29 nm Em: 628/32 nm) of EVOS FL Auto 2 Cell Imaging System, keeping the cells at optimal growing conditions in the incubation space while taking images (5% CO_2_, 37°C, >80% humidity). Images were captured using the 20× lens objective, taking nine images per well to ensure accurate depictions of the whole well. These were analyzed via Celleste image analysis software, where the total number of effector cells and target cells engulfed by effector cells for each treatment group per cell line with three technical replicates were counted. The efferocytosis index (Equation 1) was calculated as the percentage of macrophages containing at least one ingested target cell corpse:

(1) $$\begin{array}{l} Efferocytosis\;Index\\ \;=\frac{Macrophages\;with\;\geq \;1\;ingested\;target\;cell\;corpse}{Total\;macrophages}\times 100 \end{array}$$

### Efferocytosis Measurement by Image Flow Cytometry

#### Labeling of RAW ASC Target Cells

RAW-ASC cells were gathered from 80% confluent 100 mm cell culture dishes by scraping and rinsing with PBS into a 50 ml conical centrifuge tube and centrifuged for 8 min at 600 × g. The cells were then washed with 10 ml of PBS and centrifuged again to remove trace amounts of FBS from their initial media suspension. The cells were suspended in RPMI 1640 with 1% Pen-Strep and the cell density was adjusted to 1 × 10^6^ cells/ml and then 1 μl of CFSE green was added per 1 ml of the cell suspension. The cells were then incubated for 30 min protected from light at 37°C with agitation every 10 min to ensure labeling was occurring evenly throughout the media. After incubating, the cells were washed with 5× the labeling volume with complete RPMI media. The cells were then centrifuged at 220 × g for 8 min and resuspended in serum deprived RPMI media. The cell density was adjusted to 2 × 10^5^ cells/ml and plated onto, clear-bottom, 6-well plate at 4 × 10^5^ cells/well to adhere overnight. The next day, effector cells were then examined under the EVOS fluorescent microscope (Green, Ex: 470/22 Em: 525/50) to confirm the labeling process and incubated for 24 h with DHA or Veh in serum-deprived medium as described above. Death was then induced in target cells by treatment with 50 μg/ml cSiO_2_ for 16 h to generate cell corpses in the plate. cSiO_2_-induced death was assessed using microscopic observation (morphology and AO/PI staining), and cell viability assessment with MTS as described above.

#### Effector Cell Labeling

MPI cells were gathered from 80% confluent 100 mm cell culture dishes by scraping and rinsing with PBS into a 50 ml conical centrifuge tube and centrifuged for 8 min at 220 × g. The cells were then washed with 10 ml of PBS and centrifuged again to remove trace amounts of FBS from their initial media suspension. The cells were then suspended in complete RPMI and the cell density was adjusted to 5 × 10^5^ cells/ml. Two microliters of CytoTell™ Blue (AAT Bioquest, Sunnyvale, CA) from 500× stock was added per 1 ml of the cell suspension. The cells were then incubated for 30 min protected from light at 37°C; the tube was agitated every 10 min to ensure even labeling. After incubating, the cells were washed with 5× the labeling volume with complete RPMI. The cells were then centrifuged at 220 × g for 8 min and resuspended in complete RPMI with GM-CSF. The cell density was adjusted to 2.2 × 10^5^ cells/ml and cells plated onto 100 mm culture dishes to adhere overnight. The next day, effector cells were examined under the EVOS fluorescent microscope using the GFP light cube (Green, Ex: 470/22 Em: 525/50) to confirm labeling and incubated for 24 h with DHA or Veh in serum-deprived medium as described above. After DHA pre-treatment, MPI cells were collected by scraping and rinsing with PBS into a 50 ml conical centrifuge tube and resuspended in 1 ml of serum deprived RPMI MPI media with 60 ng/ml of GM-CSF at 2 × 10^5^ cells/ml.

#### Efferocytosis Assessment by Imaging Flow Cytometry

2 × 10^5^ cells of effector cells were added to the target cells at 1:2 ratio (effector: target) and then centrifuged at 500 × g for 3 min and efferocytosis measured over time. Efferocytosis was examined at time points of 1, 8, and 12 h after co-culturing of target and effector cells that were pre-incubated with either Veh or DHA. Three technical replicates were used for each treatment condition. Following the co-culturing, both effector and target cells were collected into a 50 ml conical centrifuge tube by gentle scraping and washing with DPBS and centrifuged for 10 min at 220 × g. Cells were fixed in 4% paraformaldehyde for 15 min and washed twice by centrifuging and resuspending with DPBS. Washed cells were resuspended in 0.5 ml of DPBS.

Cells were visualized for images and the efferocytosis index measured using an Image Stream MKII imaging flow cytometer (Luminex Corporation, Seattle, WA) housed in the Luminex Corporation in Seattle, WA. For each sample 10,000 images of 60× magnification in the bright field channel and the fluorescence emission channels 7 (CytoTell Blue) and 2 (CSFE) were simultaneously acquired with 50 mW 405 and 10 mW 488 laser powers. Unlabeled cells as well as cells labeled with only one fluorochrome were also acquired with the same laser setting for making the spectral compensation matrix that was applied to each of the experimental files during the image analysis with the IDEAS software. The efferocytosis index was calculated as the percentage of MPI macrophages containing at least one ingested target cell corpse (Equation 1).

### Data Analyses

For all cell death, caspase activity, and IL-1β release assays, two-way analysis of variance (ANOVA) was performed in GraphPad software (GraphPad, San Diego, CA) followed by Tukey's *post hoc* test for pairwise comparisons. If groups failed to pass at least one of normality tests (Anderson-Darling, D'Agostino-Pearson omnibus, Shapiro-Wilk, Kolmogorov-Smirnov) for Gaussian distributions, data were transformed to log_2_ format, and two-way ANOVA followed by Tukey's *post hoc* was performed in GraphPad software. Student's *t*-tests were used to compare two groups when applicable. For other *in vitro* assays, comparison of multiple groups was accomplished using a one-way ANOVA, and comparison of individual groups was accomplished using Tukey's test using GraphPad software. In all experiments, *p* < 0.05 was taken to be significant.

## Results

### DHA Supplementation Protects Against cSiO_2_-Induced Death in Three Macrophage Models

**Figure 1:**
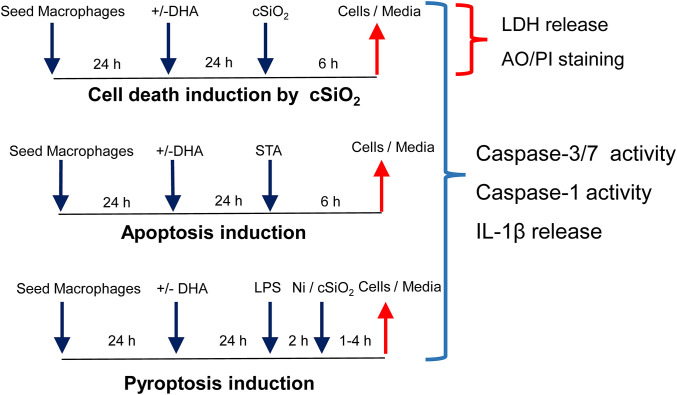
Experimental design for determining how DHA supplementation influences cSiO_2_-induced cell death, apoptosis, and pyroptosis in macrophage models. Workflow for experiments shown in Figures 2, 3. Macrophage models included (1) alveolar macrophages (AM) from NZBWF1 female mice, (2) Max Planck Institute (MPI) cells, self-renewing alveolar macrophage-like cells derived from fetal livers of C57BL/6 mice, and (3) RAW 264.7 macrophages stably transfected with ASC (RAW-ASC). cSiO_2_ has previously been shown to cause death in macrophages by apoptosis, pyroptosis, and necrosis. Apoptosis was induced by staurosporine (STA) and pyroptosis by LPS and nigericin (Ni).

**Figure 2:**
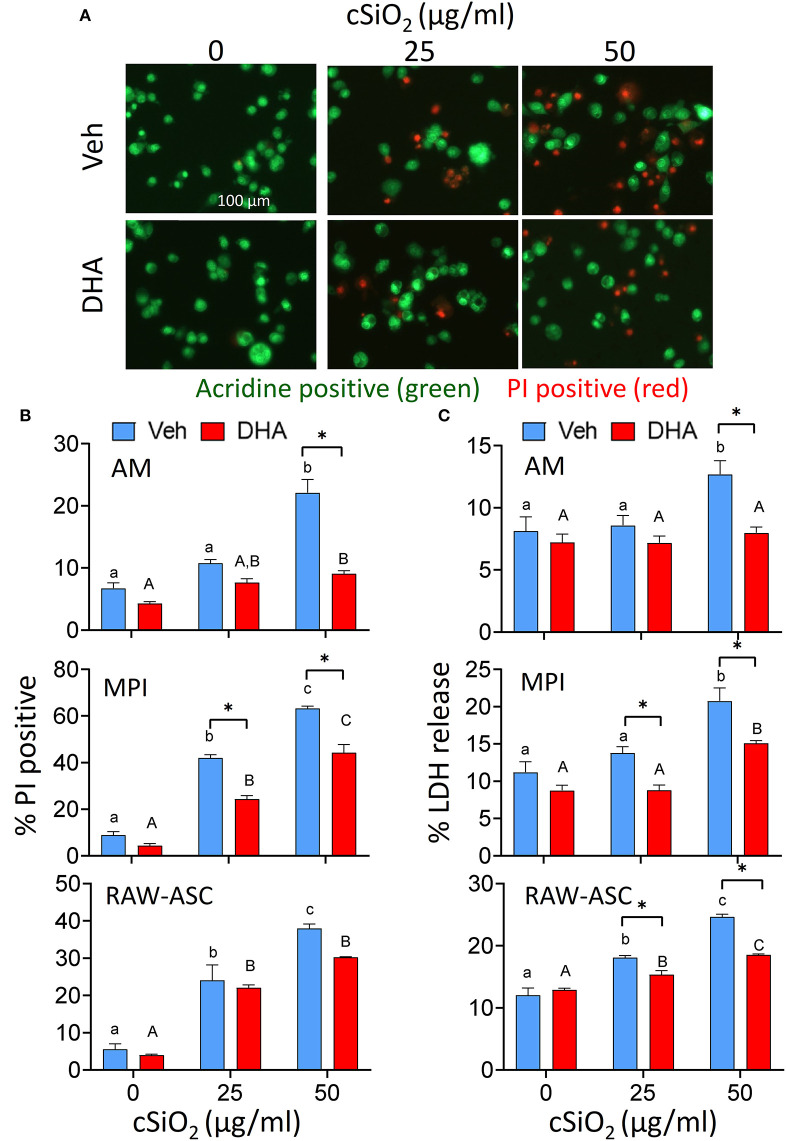
DHA supplementation protects against cSiO_2_-induced cell death in all macrophage models. AM were incubated in serum-deprived RPMI containing DHA (25 μM) or Veh for 24 h. Cells were treated with 0, 25, or 50 μg/ml cSiO_2_ for 6 h and analyzed for percentage of propidium iodide (PI) positive dead cells and LDH release. **(A)** Fluorescence microscopy of live nucleated cells stained with acridine orange (green) and dead nucleated cells stained with PI (red). Red staining confirms AM death after cSiO_2_ exposure. Images were taken at 20× magnification and a representative portion of the image is shown. DHA pre-treatment significantly inhibits cell toxicity in AM, MPI, RAW-ASC cells at the high cSiO_2_ dose (50 μg/ml) in terms of both **(B)** dead cell percentage via AO and PI staining and **(C)** LDH release. Triton X-100 was used as a positive control for lytic cell death in the LDH assay. Data presented as mean ± SEM, *n* = 3. Lowercase letters indicate significant (*p* < 0.05) differences in cSiO_2_-induced cell death within Veh supplemented group and uppercase letters indicated cSiO_2_-induced cell death within DHA-supplemented groups, as determined by two-way ANOVA followed by Tukey's *post hoc* test. Significant differences within the Control or cSiO_2_ treatment groups from Veh to DHA supplementation are represented by asterisks (*p < 0.05). Assays are representative of three independent experiments.

The effects of DHA at a physiologically relevant concentration (25 μM) on cSiO_2_-induced cell death were assessed in three different macrophage models using the experimental design depicted in Figure 1. cSiO_2_ at 50 μg/ml significantly induced death of primary AMs compared to Veh-treated cells as reflected by increased PI staining (Figures 2A,B) and LDH release (Figure 2C). These responses were significantly inhibited by inclusion of DHA. MPI cells were more sensitive to cSiO_2_'s cytotoxic effects with concentrations of 25 and 50 μg/ml inducing significantly increases in PI positive cell corpses (Figure 2B and Supplementary Figure 1A) and similar trends observed for LDH responses (Figure 2C). DHA pre-incubation significantly suppressed these responses (Figures 2B,C). RAW-ASC cells were also sensitive to cSiO_2_'s cytotoxic effects with concentrations of 25 and 50 μg/ml again significantly eliciting cell death as compared to Veh-treated cells in PI positive cell corpses (Figure 2B and Supplementary Figure 1B) and LDH release (Figure 2C). DHA pre-incubation significantly suppressed cSiO_2_-induced LDH responses (Figure 2C) with a similar trend observed for PI positive cell corpses.

### DHA Supplementation Suppresses Caspase-3/7 Activation by cSiO_2_ Alone in All Macrophage Models

**Figure 3:**
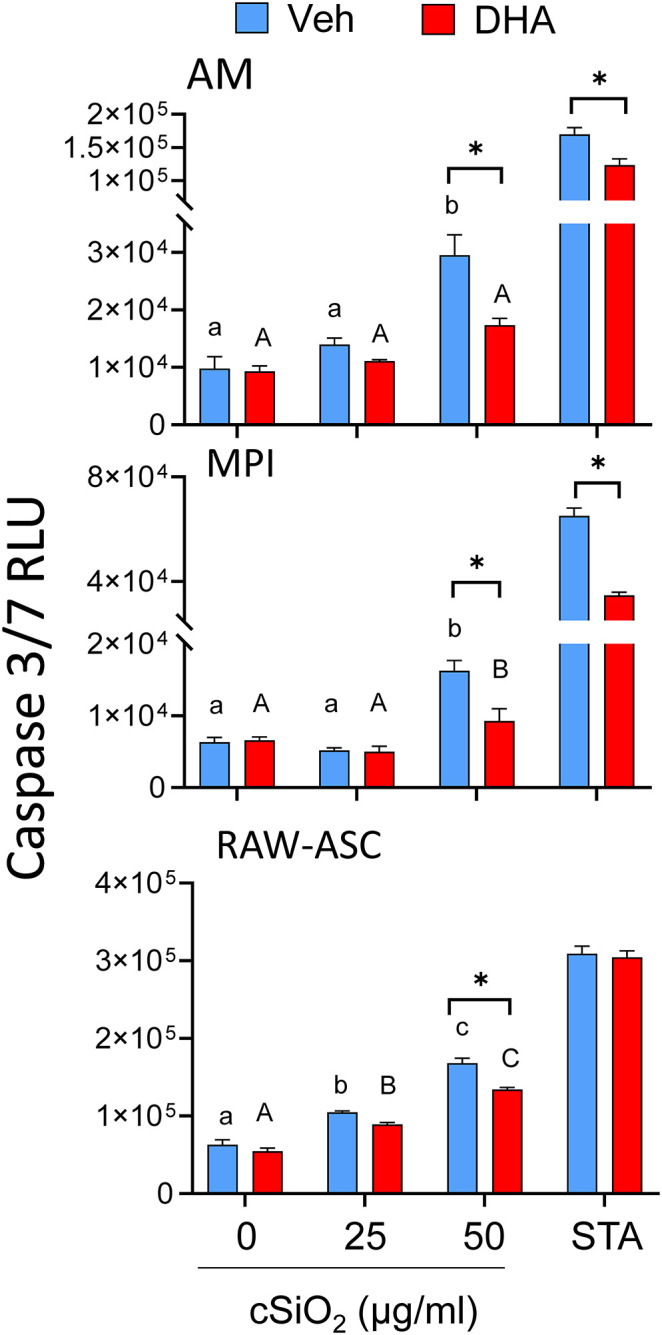
DHA supplementation suppresses cSiO_2_-induced caspase-3/7 activation in all macrophage models. AM, MPI, and RAW-ASC cells were incubated in serum-deprived RPMI containing DHA (25 μM) or Veh for 24 h. Cells were then treated with 0, 25, or 50 μg/ml cSiO_2_ for 6 h and caspase-3/7 assessed using the Caspase-Glo® 3/7 assay. Data presented as mean ± SEM, *n* = 3. Significant differences between Veh and DHA within the cSiO_2_-treated and positive control group (STA) are represented by asterisks (*p < 0.05); lowercase letters indicate significant (*p* < 0.05) differences in cSiO_2_-induced caspase-3/7 activation within Veh supplemented group and uppercase letters indicate cSiO_2_-induced caspase-3/7 within DHA-supplemented groups, as determined by two-way ANOVA followed by Tukey's *post hoc* test. Assays are representative of three independent experiments.

cSiO_2_ alone at 50 μg/ml significantly induced caspase-3/7 activity in all three macrophage models and these responses were significantly decreased when cultures were pre-incubated with DHA (Figure 3). Similar responses were observed for staurosporine, a positive control for caspase-3/7-dependent apoptosis. Thus, cSiO_2_ alone potentially induces macrophage death in part via caspase-3/7-dependent apoptosis which was inhibited by DHA.

### cSiO_2_ Alone Induces Limited Caspase-1 Activation but Not IL-1β Release in MPI and RAW-ASC Cells Without LPS Priming

cSiO_2_ at 50 μg/ml significantly induced very limited caspase-1 activation but no IL-1β release in MPI and RAW-ASC cells (Supplementary Figures 2, 3). LPS priming modestly enhanced cSiO_2_-induced caspase-1 activation and elicited robust IL-1β release. DHA pre-incubation significantly suppressed caspase-1 responses in MPI but not RAW-ASC cells and suppressed significantly IL-1β release in both primed MPI and RAW-ASC cells. Likewise, treatment with LPS plus nigericin, a positive control for caspase-1 and inflammasome-induced pyroptosis, induced caspase-1 activation and IL-1β release in these cells; both responses were inhibited by DHA treatment. Altogether, the results suggest that inflammasome activation is unlikely to play a critical role in the induction of cell death in unprimed macrophages treated with cSiO_2_ alone.

### MPI Cells Rapidly Engulf Cell Corpses of cSiO_2_-Treated, Apoptotic, Pyroptotic, or Necrotic RAW-ASC Target Cells

**Figure 4:**
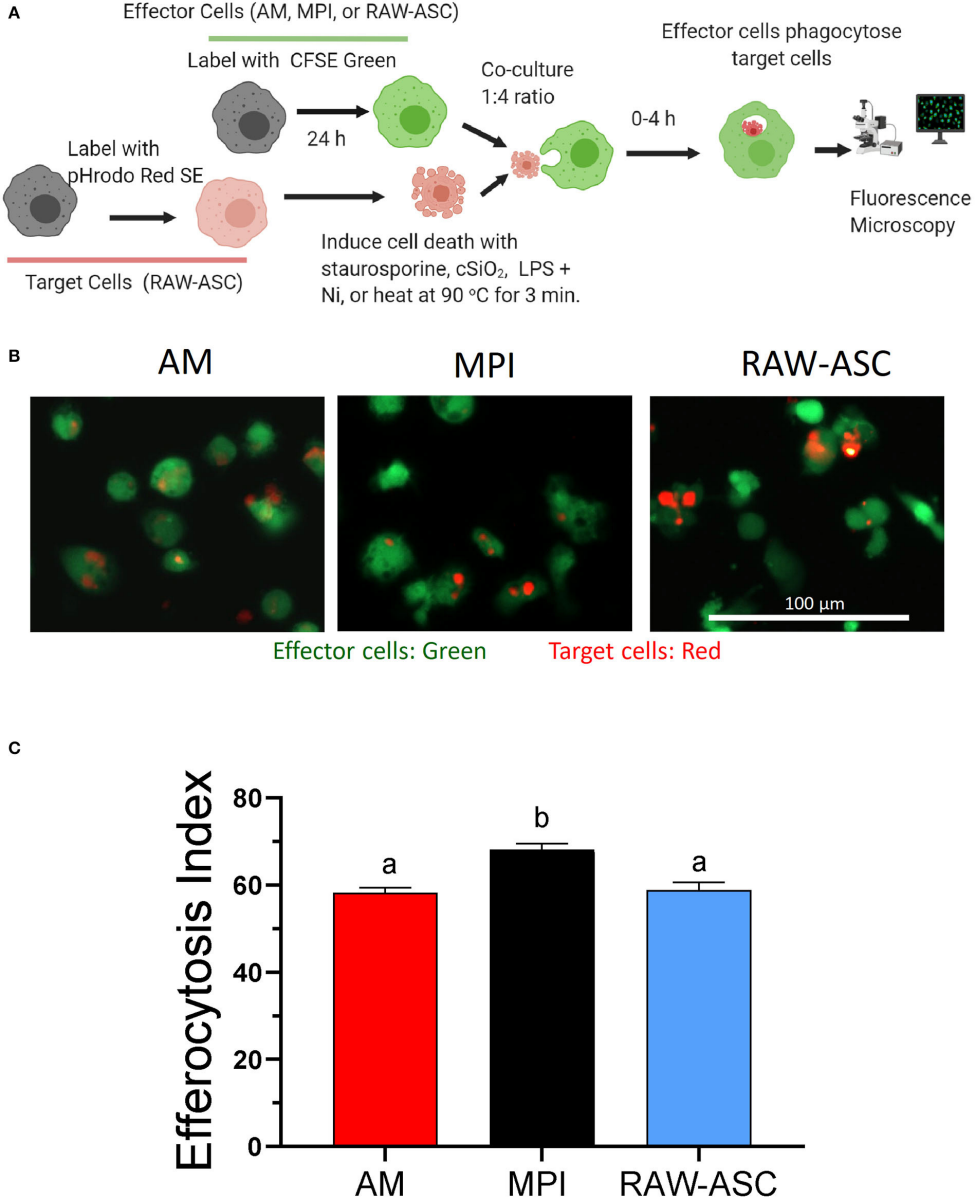
AM, MPI, and RAW-ASC cells are similarly capable of efferocytosis. **(A)** Experimental workflow for efferocytosis assay for Figures 4, 5. Death was induced in pHrodo Red SE-labeled RAW-ASC cells (target cells). Resultant cell corpses were then incubated for 1 h with CFSE-labeled macrophages (AM, MPI, and RAW-ASC) cells. Efferocytosis was assessed using an EVOS FL-2 microscope. *Created with BioRender.com*. **(B)** Representative photomicrographs demonstrating CFSE-labeled AM, MPI, and RAW-ASC effector cells (green) engulfing apoptotic RAW-ASC target cells (red) induced by staurosporine treatment. Images were taken at 20× magnification and a representative portion of the image is shown. **(C)** The efferocytosis index was calculated by analyzing merged images using Celleste™ image analyzing software. The bar plot shows the efferocytosis index of AM, MPI and RAW-ASC with apoptotic RAW-ASC cells (means ± SEM, *n* = 3). Different letters indicate significant differences between treatment groups (*p* < 0.05). Results are representative of three independent experiments.

The relative capacities of the three models to phagocytose cell corpses were compared by CFSE and pHrodo Red SE labeling of effector and target cells, respectively (Figure 4A). Fluorescent microscopy revealed that, when co-cultured and labeled under identical conditions, AM, MPI cells, and RAW-ASC models demonstrated rapid phagocytosis of apoptotic RAW-ASC cell corpse targets induced by staurosporine with efferocytosis indexes of 58, 68, and 59, respectively (Figures 4B,C). Based on these findings, MPI and RAW-ASC cells were selected as effectors and targets, respectively, for further investigating DHA's effects on efferocytosis.

**Figure 5:**
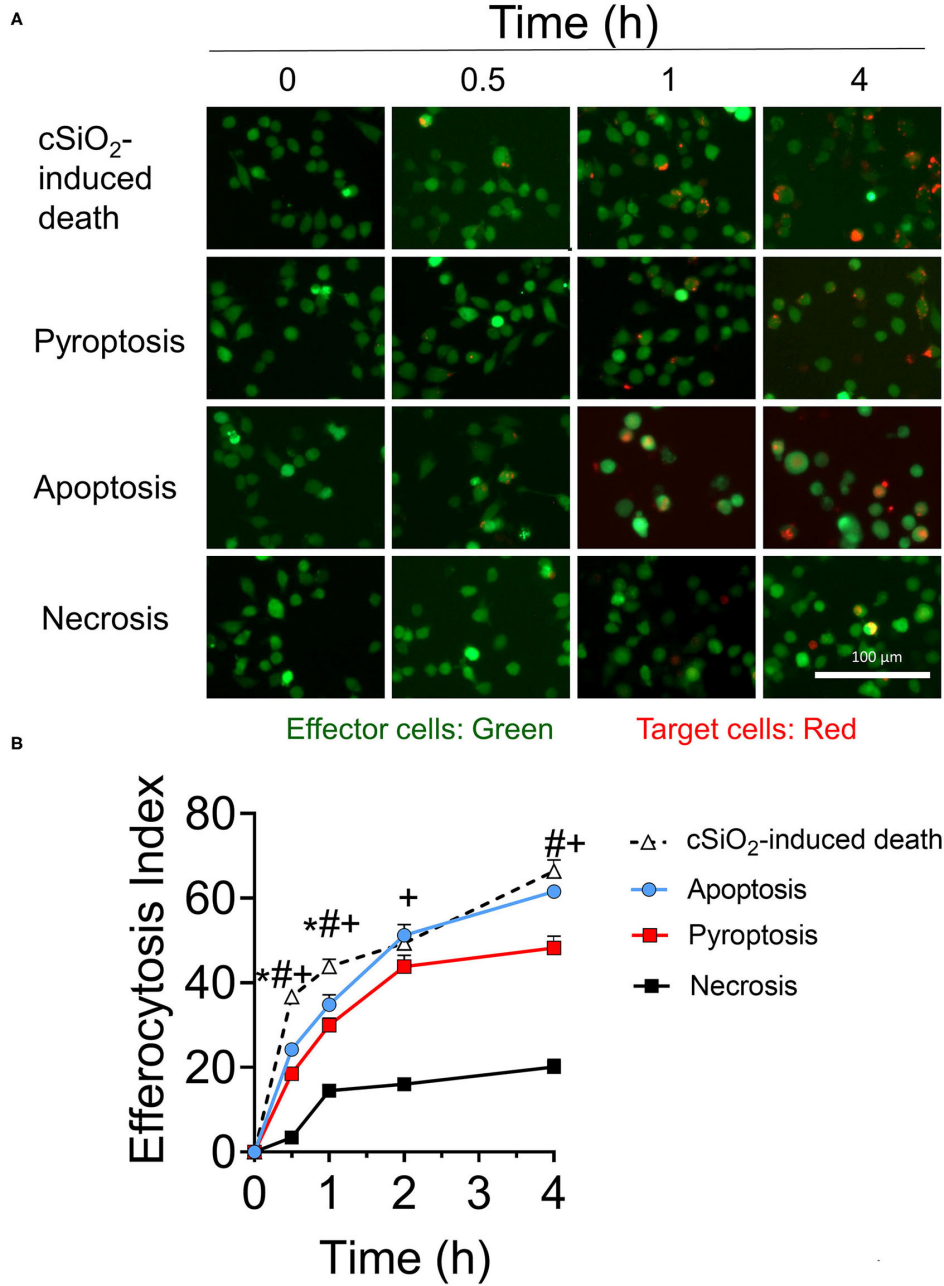
MPI cells engulf cell corpses of cSiO_2_-treated, pyroptotic, apoptotic, and necrotic RAW-ASC target cells. **(A)** Representative fluorescence photomicrographs showing CFSE-labeled MPI cells (green) engulfing pHrodo Red SE-labeled RAW-ASC target cells (red) killed by four methods: cSiO_2_ treatment, apoptosis (staurosporine), pyroptosis (LPS followed by nigericin), necrosis (held at 95°C for 3 min). **(B)** The efferocytosis index was calculated by analyzing merged images using Celleste™ image analysis software. Images were taken at 20× magnification and a representative portion of the image is shown. The line plot shows the efferocytosis index (mean ± SEM, *n* = 3) over time of MPI cells with target cells induced by the different treatments. One-way ANOVA was used to compare experimental groups at selected time points followed by *post hoc* Tukey's multiple comparison test. Data are mean ± SEM. Symbols indicate a significant difference (*p* < 0.05) as follows: *for cSiO_2_-induced death vs. apoptosis; ^#^for cSiO_2_-induced death vs. pyroptosis; and ^+^for cSiO_2_-induced death vs. necrosis. Similar results were obtained in two independent experiments.

RAW-ASC target cell corpses were generated by different cell death mechanisms including cSiO_2_-induced cell death (Supplementary Figure 4), apoptosis (Supplementary Figure 5), pyroptosis (Supplementary Figure 6), and necrosis and their engulfment by MPI effector cells evaluated over time. Overall, most efferocytosis by MPI cells of cSiO_2_-treated, apoptotic, pyroptotic, and necrotic target cells occurred within 2 h and largely leveled off by 4 h (Figures 5A,B). Efferocytosis indexes for cSiO_2_-treated, apoptotic, and pyroptotic corpses at 2 h were 49 ± 12, 51 ± 13, and 44 ± 7 (mean ± SD), respectively, and thus were comparable. Efferocytosis of necrotic corpses also was evident by 2 h but was less efficient with an efferocytosis index of 16 ± 2.

### The Efferocytosis Index Is Increased When Target Cells (RAW-ASC Cells) Are Pre-incubated With DHA

**Figure 6:**
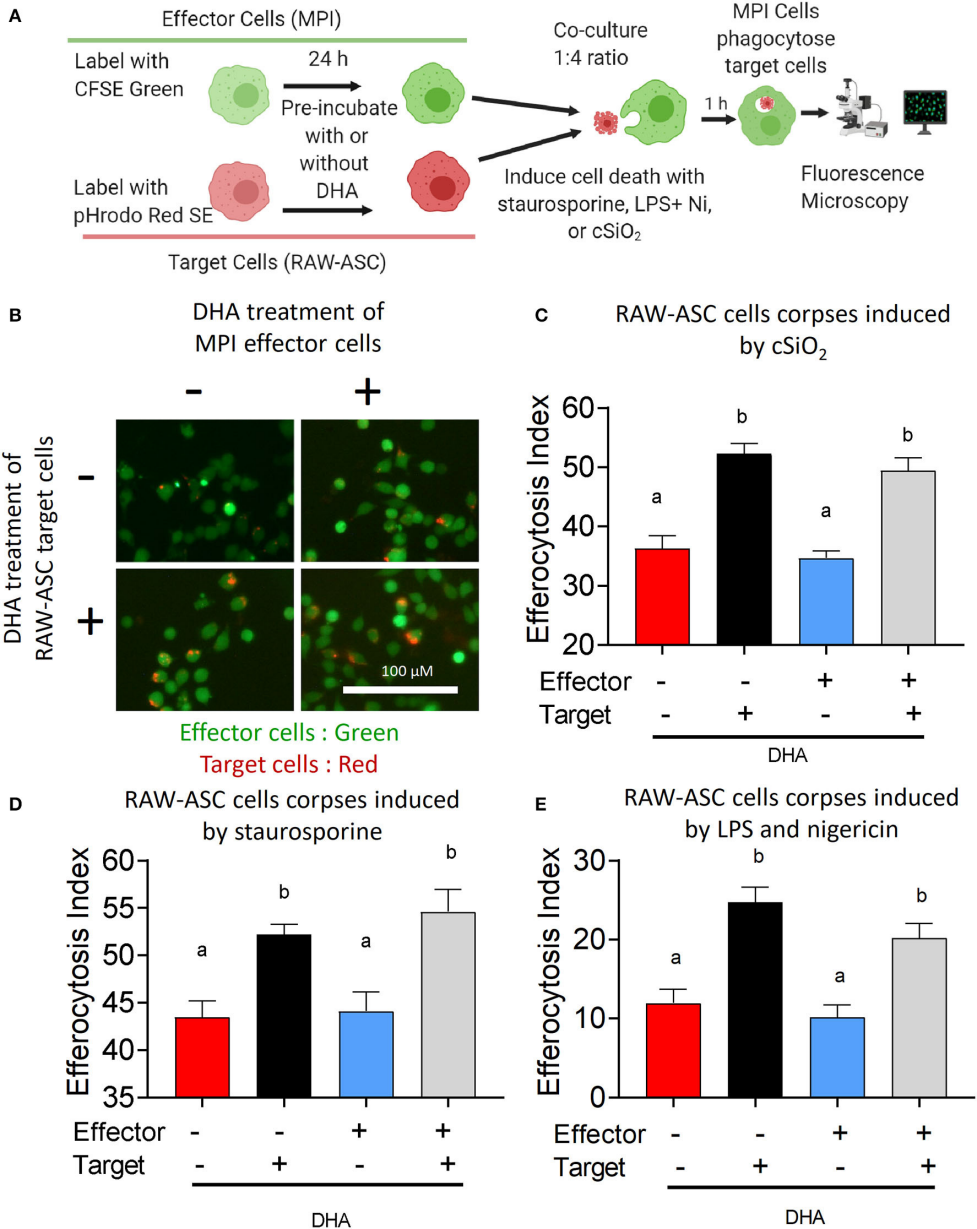
DHA enhances efferocytotic activity of MPI effector cells when RAW-ASC target cells are pre-incubated with DHA. **(A)** Experimental workflow for Figure 6 and Supplementary Figure 7. pHrodo Red SE-labeled RAW-ASC target cells were pre-incubated with Veh or 25 μM DHA for 16 h and then death induced by treatment with cSiO_2_, staurosporine and LPS+nigericin. MPI cells were pre-treated with Veh or 25 μM DHA for 24 h and their ability to efferocytose RAW-ASC corpses assessed by fluorescent microscopy. *Created with BioRender.com*. **(A,B)** Effect of DHA pre-treatments on MPI cell engulfment of apoptotic RAW-ASC cells induced by cSiO_2_. **(B)** Merged photomicrograph images were acquired using the EVOS FL 2 microscope (20×) showing pHrodo Red SE-labeled target cells (red) engulfed by the MPI cells (green). **(C–E)** Efferocytosis indexes were calculated and plotted as bar plots (mean ± SEM). Results show that DHA pre-treatment of apoptotic RAW-ASC cells increased efferocytosis index regardless of MPI cells treatment conditions. **(C)** Effect of DHA pre-treatments on MPI cell engulfment of RAW-ASC corpses induced by cSiO_2_. **(D)** Effect of DHA pre-treatments on MPI cell engulfment of RAW-ASC corpses induced by staurosporine. **(E)** Effect of DHA pre-treatments on MPI cell engulfment of RAW-ASC cells corpses induced by LPS and nigericin. Images were taken at 20× magnification and a representative portion of the image is shown. Different letters indicate significant differences between treatment groups (*p* < 0.05). Similar results were obtained in two independent experiments.

**Figure 7:**
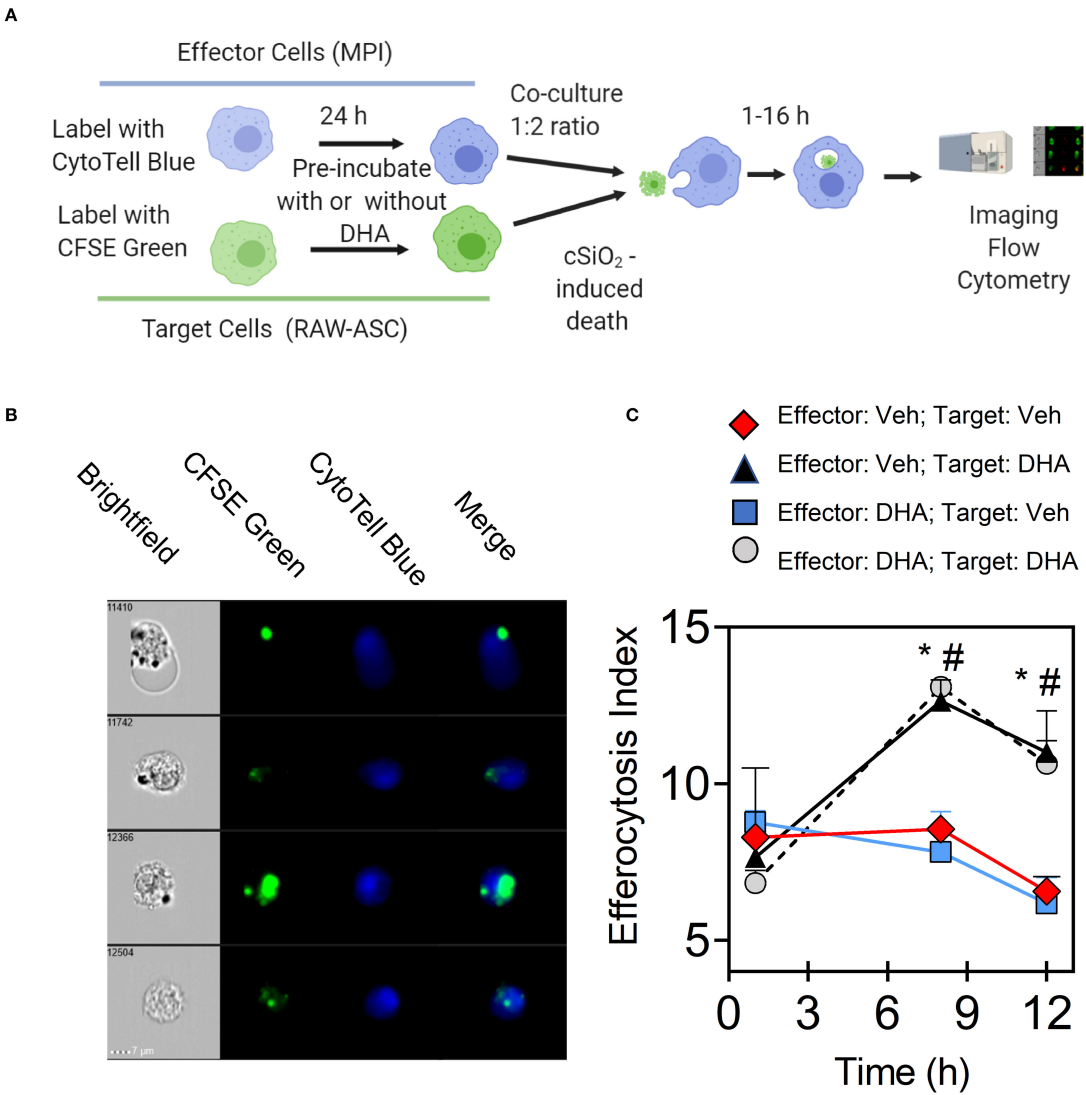
DHA enhances efferocytosis of MPI effector cells when RAW-ASC target cells are pre-incubated with DHA and death induced by cSiO_2_. **(A)** Experimental workflow for imaging flow cytometry. MPI effector cells were labeled with CytoTell blue dye and cultured overnight. Then, MPI cells were pre-incubated with Veh or 25 μM DHA for 16 h. CFSE green-labeled RAW-ASC cell corpses (target cells) were pre-incubated with Veh or DHA (25 μM) for 16 h and death induced by incubating with 50 mL cSiO_2_ for 16 h after. MPI cells were added to green-labeled RAW-ASC cell corpses (target cells) and co-incubated up to 16 h. Both MPI cells and RAW-ASC cells were collected and analyzed using Amnis® imaging flow cytometer. Created with BioRender.com. **(B)** Merged images were acquired using Amnis® imaging flow cytometer showing CFSE labeled target cells (green) engulfed by the CytoTell labeled MPI cells (blue). **(C)** Line graph shows the efferocytosis index of MPI cells (mean ± SEM) with above treatment conditions over time. Results show that DHA pre-treatment of RAW-ASC cells increased efferocytosis index regardless of MPI cells pre-treatment conditions. Symbols indicate significant difference (*p* ≤ 0.05) as follows: “*” for (Effector:Veh; Target:Veh) vs. (Effector:Veh; Target:DHA) and “^#^” for (Effector:Veh;Target:Veh) vs. (Effector:DHA;Target:DHA). Similar results were obtained in two independent experiments.

The effects of DHA supplementation of RAW-ASC target and/or MPI effector cells on efferocytosis were evaluated as depicted in Figure 6A. When RAW-ASC target cells were pre-incubated with DHA and then killed with cSiO_2_ (Figures 6B,C), staurosporine (apoptosis) (Figure 6B and Supplementary Figure 7A), or LPS and nigericin (pyroptosis) (Figure 6C and Supplementary Figure 7B) the efferocytosis indexes were significantly higher than that for target cells pre-incubated with Veh prior to cell death treatments. In contrast, DHA pre-incubation of MPI effector cells did not affect their capacity to engulf RAW-ASC cells killed by the three treatments. Significantly enhanced efferocytosis of RAW-ASC target cells pre-incubated with DHA prior to killing with cSiO_2_ was further confirmed by imaging flow cytometry (Figure 7). Again, DHA pre-incubation of MPI effector cells did not affect their capacity to engulf RAW-ASC cells killed with cSiO_2_.

## Discussion

**Figure 8:**
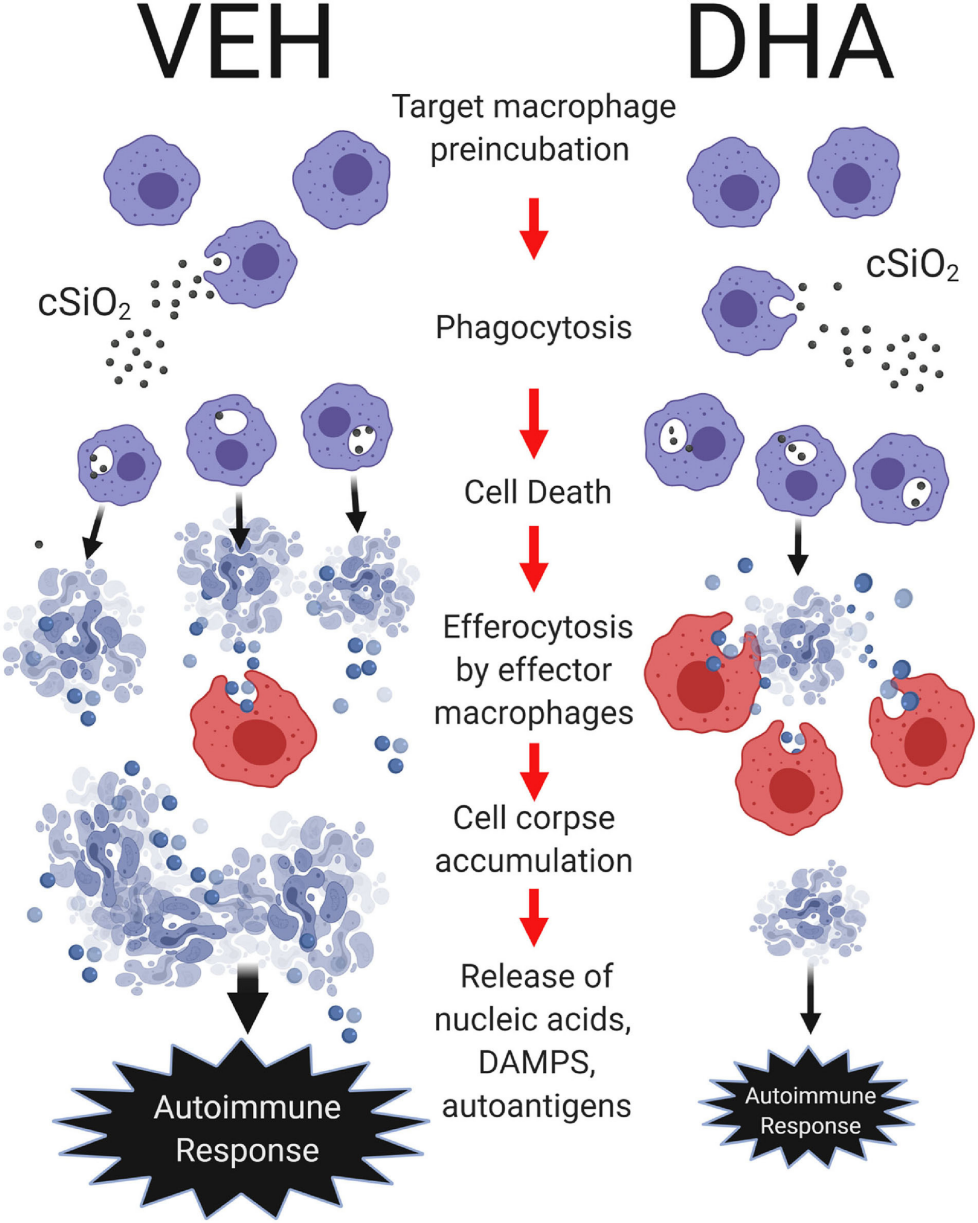
Putative model for DHA suppression of cSiO_2_-induced autoimmune response in the lung. DHA impedes cSiO_2_-induced macrophage corpse accumulation by attenuating cell death and potentiating efferocytosis. Following cSiO_2_ exposure, target macrophages phagocytose cSiO_2_, inducing cell death. Dead cells are subsequently efferocytosed by effector macrophages. Pre-incubation of target macrophages with DHA suppresses cSiO_2_-induced cellular corpse accumulation by inhibiting cell death and enhancing efferocytosis as compared to pre-incubation of target macrophages with vehicle (VEH). In the lung, heightened accumulation of cell corpses and their secondary necrosis might result in aberrant release of nucleic acids, damage-associated molecular patterns (DAMPs), and autoantigens that collectively drive robust local and systemic autoimmune responses. *Created with BioRender.com*.

cSiO_2_ clearance from the lung is very slow (48) and the persistent presence of this particle drives a vicious cycle in AMs involving phagocytosis of SiO_2_, cell death, and reemergence of free SiO_2_. Inadequate efferocytosis could result in the accumulation of autoantigen-rich cell corpses in the lungs thereby contributing to cSiO_2_ triggering of murine lupus flaring (49). The goals of this *in vitro* investigation were to determine how DHA influences both cSiO_2_-induced macrophage death and efferocytosis of cell corpses. Several novel findings were made. First, cSiO_2_-induced cell death in AM, MPI, and RAW-ASC macrophage models. Death was associated with caspase-3/7 activation that was suggestive of apoptosis. Second, DHA pre-treatment suppressed cSiO_2_-induced macrophage death and caspase-3/7 activation in all three models. Third, using the pH-sensitive dye pHrodo Red SE in conjunction with real-time fluorescence microscopy, it was demonstrated that all three macrophage models could efferocytose apoptotic RAW-ASC cells. Fourth, MPI effector cells could efferocytose cSiO_2_-killed, apoptotic, pyroptotic, and necrotic RAW-ASC cells. Finally, DHA pre-incubation of RAW-ASC target cells prior to death induction by staurosporine, LPS and nigericin, or cSiO_2_ significantly enhanced efferocytosis by MPI effector cells; however, DHA pre-incubation of MPI effector cells did not influence engulfment of RAW-ASC targets. DHA's capacity to suppress macrophage death and enhance removal of resulting corpses by efferocytosis as depicted in Figure 8 might be critical to ameliorative effects of this ω-3 PUFA in cSiO_2_-triggered murine lupus.

Following airway exposure to cSiO_2_, AMs phagocytose the particles in an effort to clear them from the lung. In an elegant single cell study employing the MH-S AM model and similar cSiO_2_ concentrations as used in this investigation, Joshi and Knecht (16) found that ~85% of exposed macrophages die by apoptosis and the remaining 15% die by necrosis. The apoptotic pathway sequentially involves phagolysosome permeabilization, transient mitochondrial hyperpolarization, caspase-3 activation, cell blebbing, nuclear condensation, and externalization of phosphatidylserine (PS). The necrotic pathway involved mitochondrial depolarization, cell swelling, and necrosis. Consistent with the apoptosis pathway, we observed that cSiO_2_ dose-dependently activated caspase-3/7 and caused cell death in all three macrophage models. In addition to apoptosis, it is further possible that caspase-3 can cleave gasdermin E N-terminal fragment, which forms pores on the plasma membrane to mediate pyroptotic/necrotic death (50–53). Therefore, it will be desirable in future studies to investigate the extent to which caspase-3 and GSMDE mediate crosstalk among apoptotic, pyroptotic, and necrotic pathways during cSiO_2_-induced macrophage cell death.

A critical finding of this study was that DHA pre-treatment suppressed cSiO_2_-induced toxicity and caspase-3/7 activation. Previously we found that when RAW-ASC cells are incubated with 25 μM DHA, cell phospholipid membrane incorporation was similar to red blood cell membrane DHA content found *in vivo* for mice fed realistic human equivalent doses of DHA (2 and 5 g per day) (20, 23, 54). In these studies, DHA dose-dependently reduced several cSiO_2_-triggered inflammatory and autoimmune endpoints in lupus-prone NZBWF1 mice. Thus, the concentration of DHA used here is physiologically relevant, and our observations consistent with prior *in vivo* findings.

Upon DHA supplementation, we observed a decrease in caspase-3/7 activity after cSiO_2_ exposure or treatment with the positive control staurosporine, consistent with a reduction in apoptotic cell death. The effect of ω-3 PUFAs on apoptosis appears to be cell type and context dependent. It has been demonstrated in multiple types of cancer that ω-3 PUFAs can induce apoptotic cell death at concentrations similar to those employed in this manuscript (55–57). However, studies in other cell types including cardiomyocytes, neurons, monocyte-like cells, and primary monocytes show that ω-3 PUFAs in fact confer protection against apoptotic stimuli (58–60). A 2014 study reported induction of apoptosis in four human myeloma cell lines treated with 50–100 μM DHA, but no apoptosis in primary peripheral blood mononuclear cells under the same conditions (61). ω-3 PUFAs have been shown to reduce oxidative stress *in vitro, in vivo*, and in human clinical trials (62), which may be a mechanism by which DHA attenuates apoptotic cell death. In support of this, neural progenitor cells isolated from *fat-1* mice, which endogenously produce higher levels of ω-3 PUFAs, were protected from H_2_O_2_-induced apoptosis (63). Similarly, the observed decrease in apoptosis with ω-3-supplementation *in vivo* is associated with a decrease in reactive oxygen species (ROS) (60). Therefore, additional research is needed to clarify how ω-3 PUFAs influence oxidative stress and how these effects impact apoptosis in macrophages.

It should be further noted that cSiO_2_-exposed macrophages can also undergo NRLP3 inflammasome-dependent programmed lytic cell death via pyroptosis (64). In brief, cSiO_2_ elicits lysosomal membrane permeabilization followed by NLRP3 inflammasome oligomerization and caspase-1 activation (42, 65–67). Caspase-1 selectively cleaves pro-IL-1β to mature IL-1β and induces cell death via pyroptosis (68–72). IL-1β release and pyroptotic cell death in macrophages is mediated by gasdermin D (GSDMD), through active caspase-1's generation of the gasdermin D N-terminal fragment (GSDMD-NT), which executes these events by forming pores on the plasma membrane (68, 71). Since expression of NLRP3 requires priming with microbial ligands such as LPS or endogenous cytokines, cSiO_2_ did not induce IL-1β release in unprimed macrophages. This suggests that the particle alone did not activate NRLP3 inflammasome-directed pyroptosis. We have previously reported that priming RAW-ASC cells with LPS prior to cSiO_2_ treatment induces robust inflammasome activation, as assessed by IL-1β release, ASC oligomerization, and caspase-1 activation (23). Consistent with this possibility, we observed here that priming RAW-ASC cells and MPI cells with LPS prior to cSiO_2_ treatment induces inflammasome activation, as assessed by robust IL-1β release which was inhibitable by DHA (Supplementary Figure 3). Likewise nigericin following priming with LPS, a positive control for pyroptosis, induces robust caspase-1 activation and IL-1β release which was also inhibited by DHA pre-treatment. Thus, DHA supplementation was also effective in blocking inflammasome activation.

Efferocytosis plays a critical role in removing dead cells containing autoantigens, thus suppressing untoward innate and adaptive immune responses (73). Numerous microscopic and flow cytometric approaches have been described to measure efferocytosis *in vitro* using targets labeled with a variety of dyes (74). A common problem with many is that detection of surface-bound, non-engulfed dead cells leads to overestimation of target cell engulfment. The method used here, based on a modification of Mikasa et al. (75), obviates this problem by employing the pH-sensitive fluorescent dye pHrodo Red SE. This dye passively diffuses into cells and covalently binds to amino groups on intracellular macromolecules. Under neutral pH, pHrodo Red SE is not detectable by fluorescent microscopy or flow cytometry. Upon phagocytosis, the pH decreases due to post-phagolysosomal fusion acidification, which facilitates red fluorescent emission of pHrodo Red SE. Accordingly, while pHrodo Red SE-labeled, non-phagocytosed RAW-ASC target cells are undetectable, following engulfment, the labeled-target cells emit an intense red signal due to the acidic environment in the phagolysosomes of CFSE-labeled MPI or other macrophage effectors. Using pHrodo Red SE in conjunction with real-time fluorescence microscopy, it was demonstrated that all three macrophage models were capable of efferocytosing apoptotic RAW-ASC cells.

Poor clearance and accumulation of dead cells has been extensively reported in diseases of chronic inflammation (76, 77) and autoimmunity (78–81), suggesting that effective efferocytosis of cell death corpses from multiple death mechanisms is essential for the maintenance of tissue homeostasis (27, 82). Ingestion of apoptotic cells by phagocytes prevents release of their intracellular contents therefore precluding inflammation (19). However, if the apoptotic cells are not cleared rapidly, they proceed into secondary necrosis which releases alarmins, damage-associated molecular patterns, and self-antigens (3). Similarly, cells undergoing necrosis or pyroptosis lose membrane integrity and leak their intracellular components which can include danger signals that promote inflammation (22, 23). Therefore, clearance of both apoptotic cells and other dead cells is extremely important in retaining tissue homeostasis. We found here that MPI effector cells engulf cSiO_2_-killed, apoptotic, and pyroptotic RAW-ASC cells at a similar clearance rate. Further, we induced necrosis in RAW-ASC cells and found that the necrotic corpses could be phagocytosed by MPI cells, albeit at a lesser clearance rate than cells killed by the aforementioned death mechanisms. Consistent with this finding, it has been reported previously that heat-killed necrotic corpses were efferocytosed by macrophages or NIH3T3 cells (83, 84). Thus, the real-time fluorescence microscopy method should detect phagocytosis of RAW-ASC corpses undergoing secondary necrosis.

A critical finding was that the efferocytosis index was significantly increased only when the target cells were pre-incubated with DHA. The observation that DHA content of an apoptotic cell increases an “eat me” signal has been heretofore unreported and thus the underlying mechanisms are unclear. During pyroptosis and apoptosis, PS normally present in the inner leaflet, is revealed on the cell surface (83, 85). PS facilitates recognition by macrophages and consequent engulfment of the cell corpse. Importantly, oxidized phosphatidylserine (oxPS) on the surface of apoptotic cells shows a predilection for recognition by macrophages and consequent engulfment compared to cell corpses with PS (86–88). OxPS on the surface of apoptotic cells can be generated in a caspase-dependent manner (89), providing a death-specific marker for the PS receptors on effector cells. PS can also be oxidized in a caspase-independent manner during inflammation as a result of enhanced lipid peroxidation (90). Moreover, apoptotic cell recognition by macrophages via the CD36 scavenger receptor transpires largely through interactions with oxPS but not non-oxPS (90). Similarly, other important scavenger receptors (SR) for efferocytosis associated in apoptotic cell clearance including SR, SRB1, SRA, LOX-1, CD68, and CD14 (86, 91) also appear to selectively recognize the oxidized sn-2 acyl group where DHA binds to the glycerol structure. Of high relevance to the present investigation, Hellwing et al. (92) reported that incubation of RAW 264.7 cells with DHA increases the content of PS containing this ω-3 PUFA at the sn-2 position at the expense of PS species containing monounsaturated fatty acids. Therefore, since DHA has six unsaturated bonds that are susceptible to oxidation whereas monosaturated fatty acids have only one unsaturated double bond, pre-incubation with DHA perhaps increases the levels of oxidizable PS molecular species in the cell membrane of the RAW-ASC target cells. Further extensive investigation is needed to clarify if this putative mechanism is responsible for increased efferocytosis of target cells pre-incubated with DHA.

DHA and its lipid metabolites might also indirectly influence efferocytosis. Specifically, the engulfment of dead cell corpses might bring in large amounts of cellular lipids, including DHA and its specific pro-resolving metabolites (SPMs), into the intracellular compartments of the phagocyte (93–95). Some studies have shown that these internalized lipids can activate PPAR-δ receptors (93) and the nuclear receptor LXR in macrophages (94) induce expression of engulfment receptors such as Mer and C1q. In mice, genetic ablation of PPAR-δ results in impaired apoptotic cell clearance and SLE-like disease (95). In further support of this notion, DHA and its SPMs including, resolvins, protectins, and maresins, have been previously reported to enhance clearance of apoptotic cells (23–29). Additional study is therefore warranted on the mechanisms by which DHA enhances the eat-me signal in target macrophages.

A limitation of this study is the lack *in vivo* data from animal models. The association between cSiO_2_ and autoimmunity has been well-documented in several *in vivo* mouse models over the last three decades (17, 18, 21, 96, 97). These studies have clearly delineated that cSiO_2_ exposure leads to one or multiple exacerbated autoimmune conditions including an increase in pulmonary lesions along with dead cell accumulations, autoantibodies, circulation and deposition of immune complexes, and glomerulonephritis. Specifically, Holian and coworkers (18, 65) reported that autoantibodies from mice with cSiO_2_-exacerbated autoimmune responses recognize specific epitopes on apoptotic macrophages suggesting cSiO_2_ induced apoptosis of AM and accumulation of cSiO_2_-induced cell corpses may be a critical triggering autoantibody production and immune complex deposition in lupus-prone mice. Similarly, other studies showed the accumulation of apoptotic or other dead cells in the lung upon cSiO_2_ exposure using TUNEL staining (98, 99). Altered efferocytosis after cSiO_2_ inhalation could lead to an excess of uncleared apoptotic cells, which could drive subsequent increased production of autoantibodies in above *in vivo* studies. In support of this notion, cSiO_2_ inhalation significantly decreases the clearance of apoptotic cells by efferocytosis in mice (100). Nevertheless, AMs exist in a pulmonary microenvironment that contains surfactants and components of the lung lining fluid that are identified to stimulate optimum macrophage function (101–105). *Ex vivo* manipulation of AM or using AM models includes plating and incubation with apoptotic cells. These manipulations may evoke both physiological and epigenetic changes that alter efferocytosis (106–108). Therefore, to extend the translational value of the present investigation, it will be necessary in the future to determine DHA's *in vivo* effects on cSiO_2_-induced cell death and subsequent efferocytosis in the lung without *ex vivo* manipulations (109).

To summarize, DHA at a physiologically relevant concentration attenuates cSiO_2_-induced apoptotic cell death in three AM models while potentiating efferocytosis, with the net effect of reducing cell corpse accumulation (Figure 8). Reduced cell corpses could diminish the availability of nucleic acids that induce interferon-related gene responses and autoantigens that drive autoimmune progression and production of pathogenic autoantibodies. These findings provide plausible mechanisms for our prior observations that dietary DHA supplementation can suppress cSiO_2_-triggered autoimmune flaring in lupus-prone mice. To date, there are no specific pharmacological approaches for enhancing clearance of dead cells. At the translational level, our results suggest potential benefits ω-3 PUFA consumption for patients with lupus, particularly those in remission. Further insights are needed into how DHA and other ω-3 PUFAs in the membrane as well as their metabolites might influence cell death during autoimmune disease and how their presence in dying or dead cells might affect their phagocytosis by nearby macrophages.

## Data Availability Statement

The raw data supporting the conclusions of this article will be made available by the authors, without undue reservation.

## Ethics Statement

The animal study was reviewed and approved by Institutional Animal Care and Use Committee at Michigan State University.

## Author Contributions

LR: study design, data analyses/interpretation, figure preparation, manuscript preparation, investigation, and manuscript editing. PC: study design, data analyses/interpretation, and figure editing. KW: MPI cell isolation, manuscript preparation, and manuscript editing. AE and SH: lab and data analyses. MB: study design and lab analyses. MG: generation of MPI cells and RAW-ASC model. JP: planning, coordination, oversight, manuscript preparation/submission, and project funding. All authors contributed to the article and approved the submitted version.

## Conflict of Interest

The authors declare that the research was conducted in the absence of any commercial or financial relationships that could be construed as a potential conflict of interest.
