# Supplementary material for: Omega-3 Docosahexaenoic Acid (DHA) Impedes Silica-Induced Macrophage Corpse Accumulation by Attenuating Cell Death and Potentiating Efferocytosis
Source: Front Immunol. 2020 Oct 6;11:2179. doi: 10.3389/fimmu.2020.02179 (PMC7573148; doi:10.3389/fimmu.2020.02179)
Supplement: Supplementary file 1 [file Presentation_1.pdf]

Omega-3 docosahexaenoic acid (DHA) impedes silica-induced macrophage corpse accumulation by attenuating cell death and potentiating efferocytosis

Lichchavi D. Rajasinghe* [1,2], Preeti S. Chauhan* [1,2], Kathryn A. Wierenga[2,3] , Augie O. Evered[1], Shamya N. Harris, Melissa A. Bates[1,2], Mikhail A. Gavrilin[4], and James J. Pestka [1,2,5]*

<u>Supplementary Figures</u>

- Supplementary Figure 1: DHA supplementation suppresses $cSiO_2$-induced cell death in MPI cells and RAW-ASC cells. MPI cells and RAW-ASC cells were incubated in serum-deprived RPMI containing DHA (25 μM) or vehicle (Veh) for 24 h.

- Supplementary Figure 2: DHA supplementation suppresses $cSiO_2$-induced caspase-1 activation in LPS primed and unprimed MPI cells.

- Supplementary Figure 3: DHA supplementation suppresses $cSiO_2$-induced IL-1β release in LPS primed MPI and RAW-ASC cells.

- Supplementary Figure 4: Confirmation of cSiO2-induced cell death in RAW-ASC target cells. Incubation of target cells (RAW-ASC) with $cSiO_2$ produces dead cell corpses.

- Supplementary Figure 5. Confirmation of staurosporine-induced apoptotic cell death in RAW-ASC target cells.

- Supplementary Figure 6: Confirmation of pyroptotic cell death in target cells by LPS and nigericin.

- Supplementary Figure 7: DHA enhances efferocytotic activity of MPI effector cells when RAW-ASC target cells are pre-incubated with DHA.

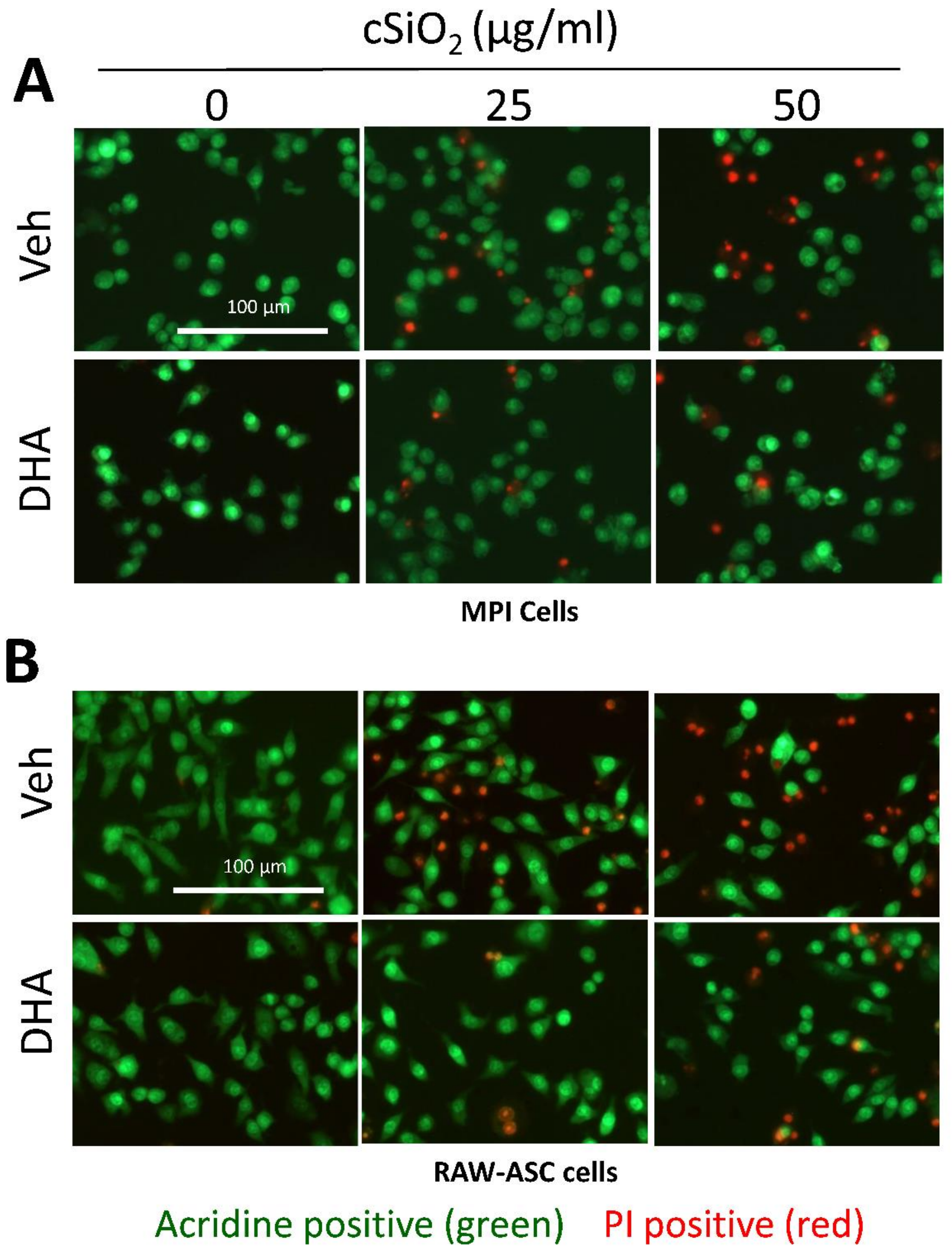

Supplemental figure 1

Supplementary Figure 1: DHA supplementation suppresses $cSiO_2$-induced cell death in MPI cells and RAW-ASC cells. MPI cells and RAW-ASC cells were incubated in serum-deprived RPMI containing DHA (25 µM) or vehicle (Veh) for 24 h. Cells were treated with 0, 25, or 50 µg/ml $cSiO_2$ for 6 h and analyzed for percentage of PI^+ dead cells. Fluorescence microscopy of live nucleated cells stained with acridine orange (green) and dead nucleated cells stained with PI (red). Red staining confirms cell death after $cSiO_2$ exposure in **(A)** MPI cells and **(B)** RAW-ASC cells. Images were taken at 20x magnification and representative of three independent experiments.

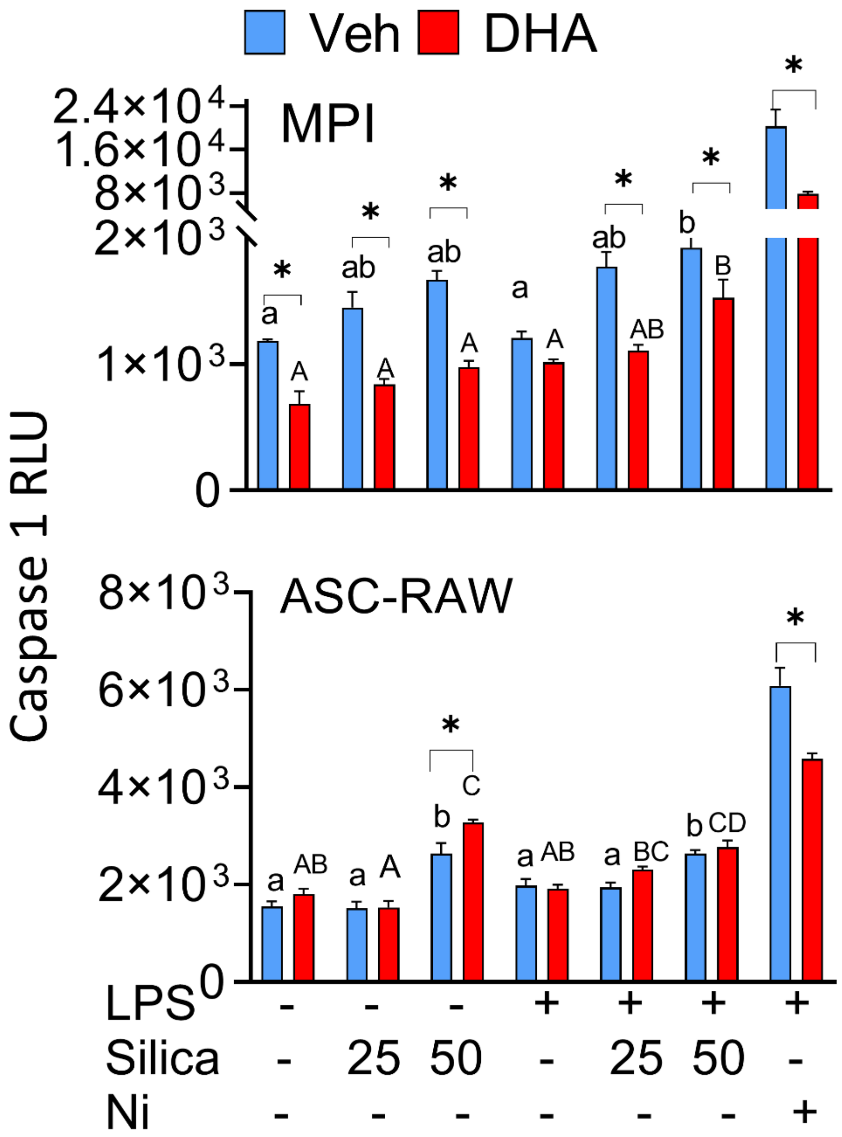

Supplemental Fig 2

Supplementary Figure 2: DHA supplementation suppresses $cSiO_2$-induced caspase-1 activation in LPS primed and unprimed MPI cells. MPI, and RAW-ASC cells were incubated in serum-deprived RPMI containing DHA (25 µM) or Veh for 24 h. Cells were then treated with 0, 25, or 50 µg/ml $cSiO_2$ for 6 h following priming with 20 ng/ml LPS or Veh for 2 h, and caspase-1 activation assessed using the Caspase-Glo® 1 assay. Data presented as mean ± SEM, n=3. Significant differences between Veh and DHA within the Control, $cSiO_2$, or nigericin treatment groups represented by asterisks, as determined by unpaired t-test ($p<0.05$); lowercase letters indicate significant differences in $cSiO_2$-induced caspase-1 activation within Veh-supplemented groups and uppercase letters indicate significant differences in $cSiO_2$-induced caspase-1 activation within DHA-supplemented groups, as determined by two-way ANOVA followed by Tukey's post hoc test ($p<0.05$). Assays are representative of three independent experiments.

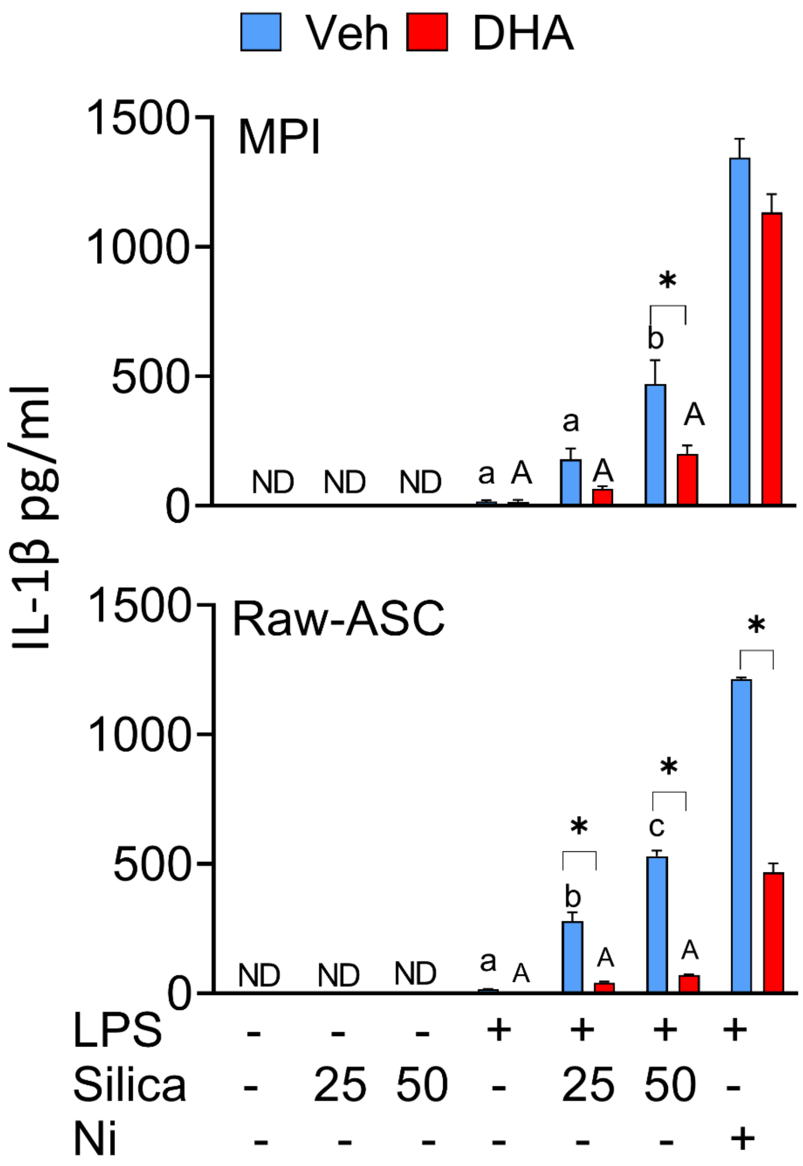

Supplemental Fig 3

Supplementary Figure 3: DHA supplementation suppresses $cSiO_2$-induced IL-1β release in LPS primed MPI and RAW-ASC cells. MPI, and RAW-ASC cells were incubated in serum-deprived RPMI containing DHA (25 µM) or Veh for 24 h. Cells were then treated with 0, 25, or 50 µg/ ml $cSiO_2$ for 6 h following priming with 20 ng/ml LPS or Veh for 2 h, and IL-1β release assessed using IL-1β/IL-1F2 DuoSet® ELISA Assay (R&D Systems). Data presented as mean ± SEM, n=3. Significant differences between Veh and DHA within the Control, $cSiO_2$, or nigericin treatment groups represented by asterisks, as determined by unpaired t-test ($p<0.05$); lowercase letters indicate significant differences in $cSiO_2$-induced IL-1β release within Veh-supplemented groups and uppercase letters indicate significant differences in $cSiO_2$-induced IL-1β release within DHA-supplemented groups, as determined by two-way ANOVA followed by Tukey's post hoc test ($p<0.05$). Assays are representative of three independent experiments.

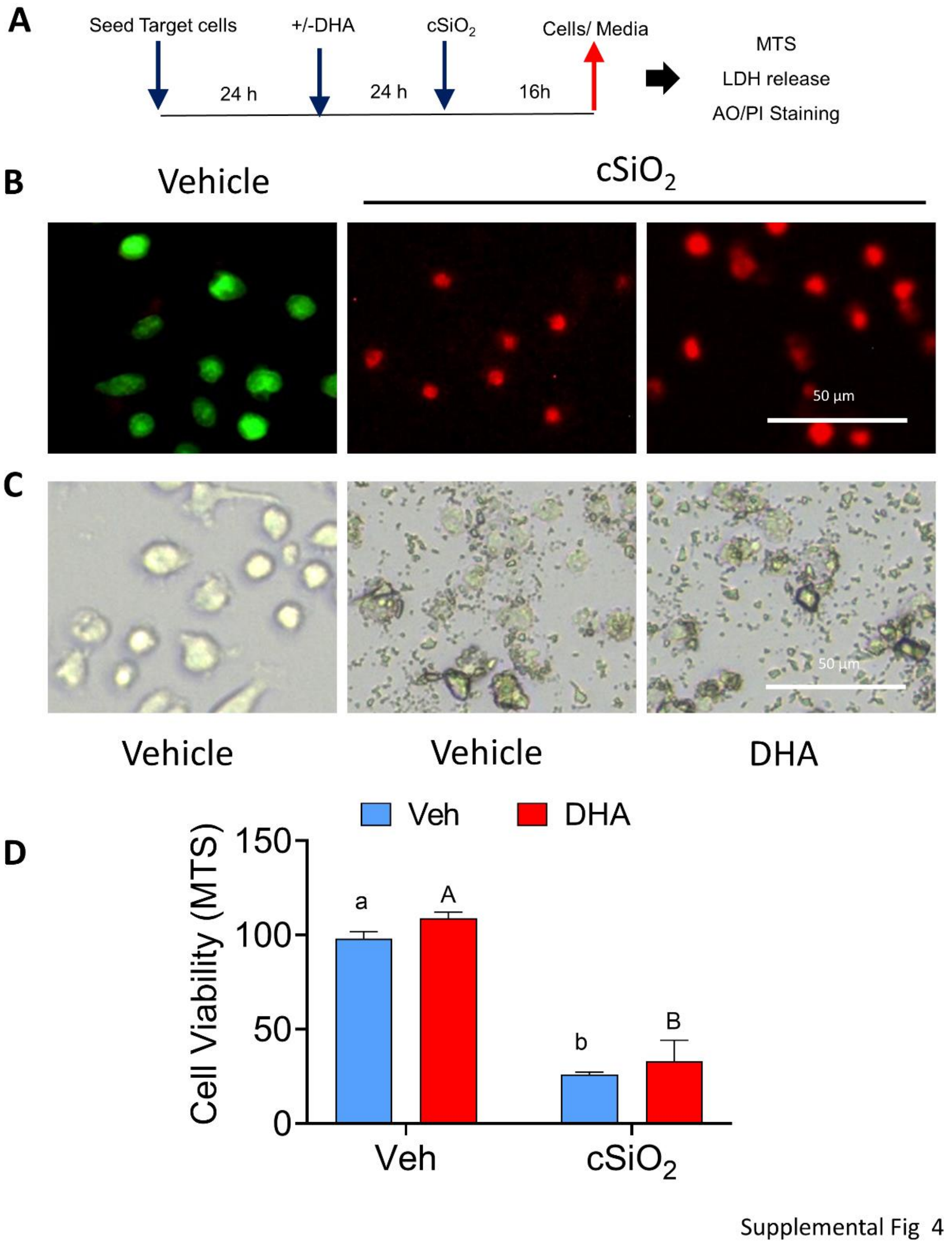

Supplementary Figure 4: Confirmation of $cSiO_2$-induced cell death in RAW-ASC target cells. Incubation of target cells (RAW-ASC) with $cSiO_2$ produces dead cell corpses. RAW-ASC cells were incubated in serum-deprived RPMI containing DHA (25 µM) or Veh for 24 h. Cells were pretreated with 50 µg/ml $cSiO_2$ for 16 h as shown in study design **(A),** and then fluorescence images **(B)** were captured with 20x magnification after labeling with AO and PI. **(C)** Bright field images were captured with 20x magnification to confirm apoptotic cell morphology. Representative bright field and fluorescence images were presented at their original size. **(D)** Percentage of viable cells (MTS assay) was measured. Data presented as mean ± SEM, n=3. Lowercase letters indicate significant differences in $cSiO_2$-induced cell death within Veh-supplemented group and uppercase letters indicate differences in $cSiO_2$-induced cell death within Veh-supplemented group, as determined by unpaired t-test (p<0.05). Assays are representative of three independent experiments.

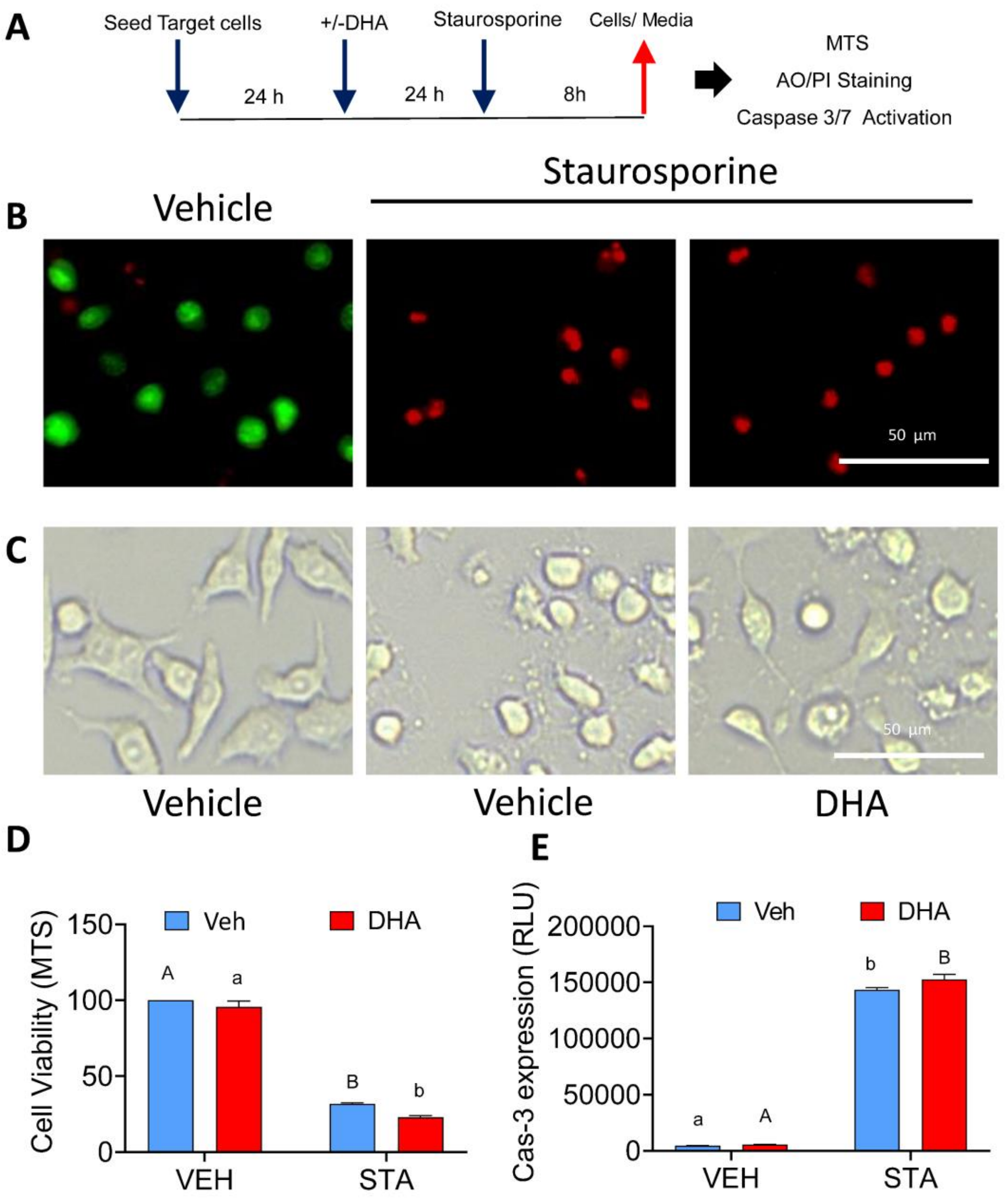

Supplementary Figure 5. Confirmation of staurosporine-induced apoptotic cell death in RAW-ASC target cells. RAW-ASC cells were incubated in serum-deprived RPMI containing DHA (25 µM) or Veh (BSA) for 24 h. Cells were treated with 0.5 µM staurosporine for 8 h as shown in study design **(A),** and then fluorescence images **(B)** were captured with 20x magnification after labeling with AO and PI. **(C)** Bright-field images were captured with 20x magnification to confirm apoptotic cell morphology. Representative potion of bright field and fluorescence images were presented at their original size. (**D)** Percentage of viable cells (MTS assay) and **(E)** release of caspase-3 was measured to confirm apoptotic markers. Data presented as mean ± SEM, n=3. Lowercase letters indicate significant differences in $cSiO_2$-induced cell death within Veh supplemented group and uppercase letters indicate significant differences in $cSiO_2$-induced cell death within DHA-supplemented groups, as determined by unpaired t-test (p<0.05). `

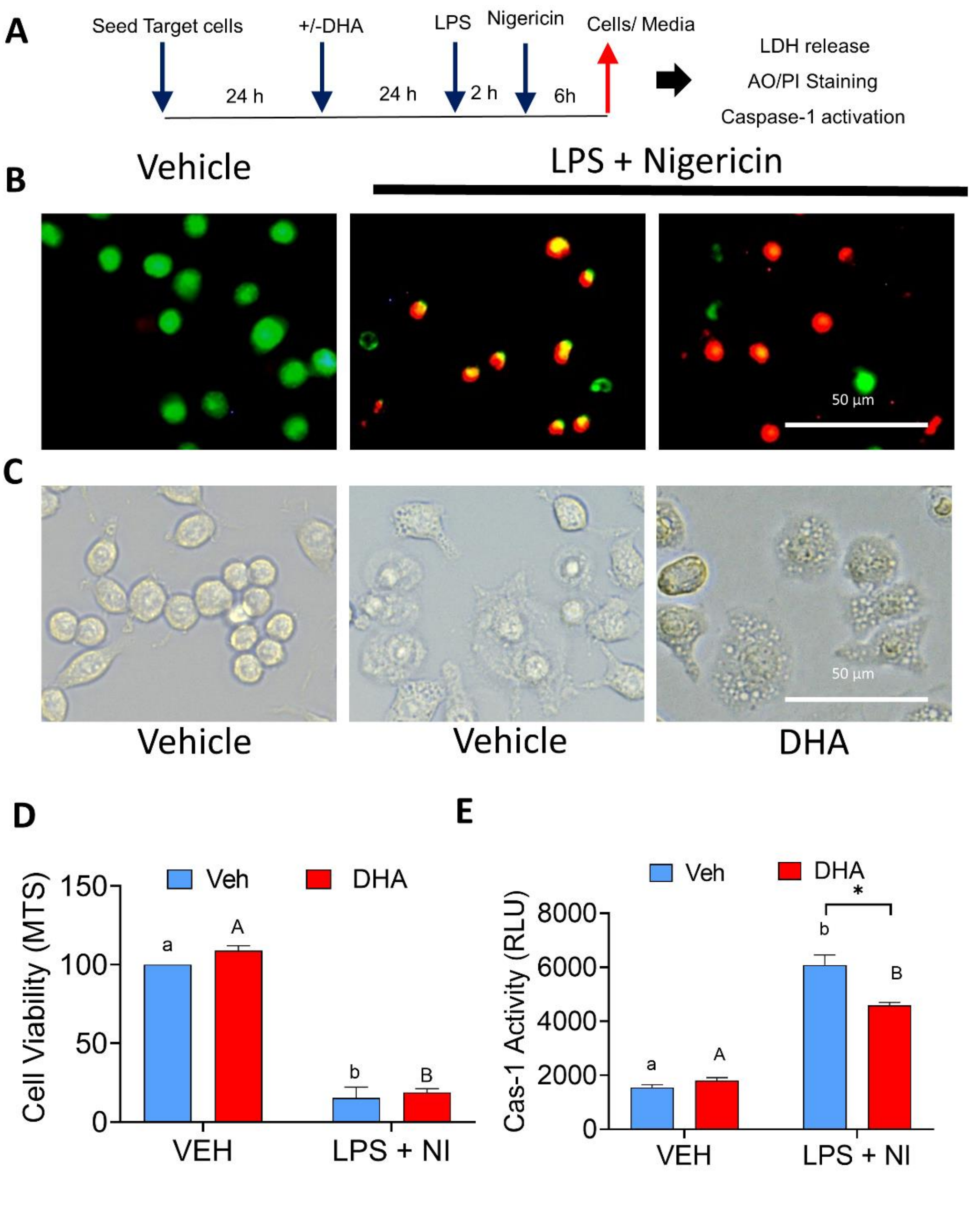

Supplemental Fig 6

Supplementary Figure 6: Confirmation pyroptotic cell death is induced in target cells by LPS and nigericin. RAW-ASC cells were incubated in serum-deprived RPMI containing DHA (0 or 25 µM) or VEH (BSA) for 24 h. Cells were pretreated with 20 ng/ml LPS for 2 h, incubated with 0.25 µM nigericin for 6h, as shown in study design **(A)**, and then fluorescence images (B) were captured with 20x magnification after labeling with AO and PI. **(C)** Bright field images were captured with 40x magnification to confirm pyroptotic cell morphology. (**D)** Percentage of viable cell (MTS assay) and **(E)** release of caspase-1 was measured. Data presented as mean ± SEM, n=3. Lowercase letters indicate significant differences in cSiO_2-induced cell death within Veh-supplemented group and uppercase letters indicate significant differences in cSiO_2-induced cell death within DHA-supplemented groups, as determined by unpaired t-test ($p<0.05$). Significant differences between DHA-supplemented and Veh-supplemented cells indicated by asterisks, as determined by unpaired t-test ($p<0.05$).

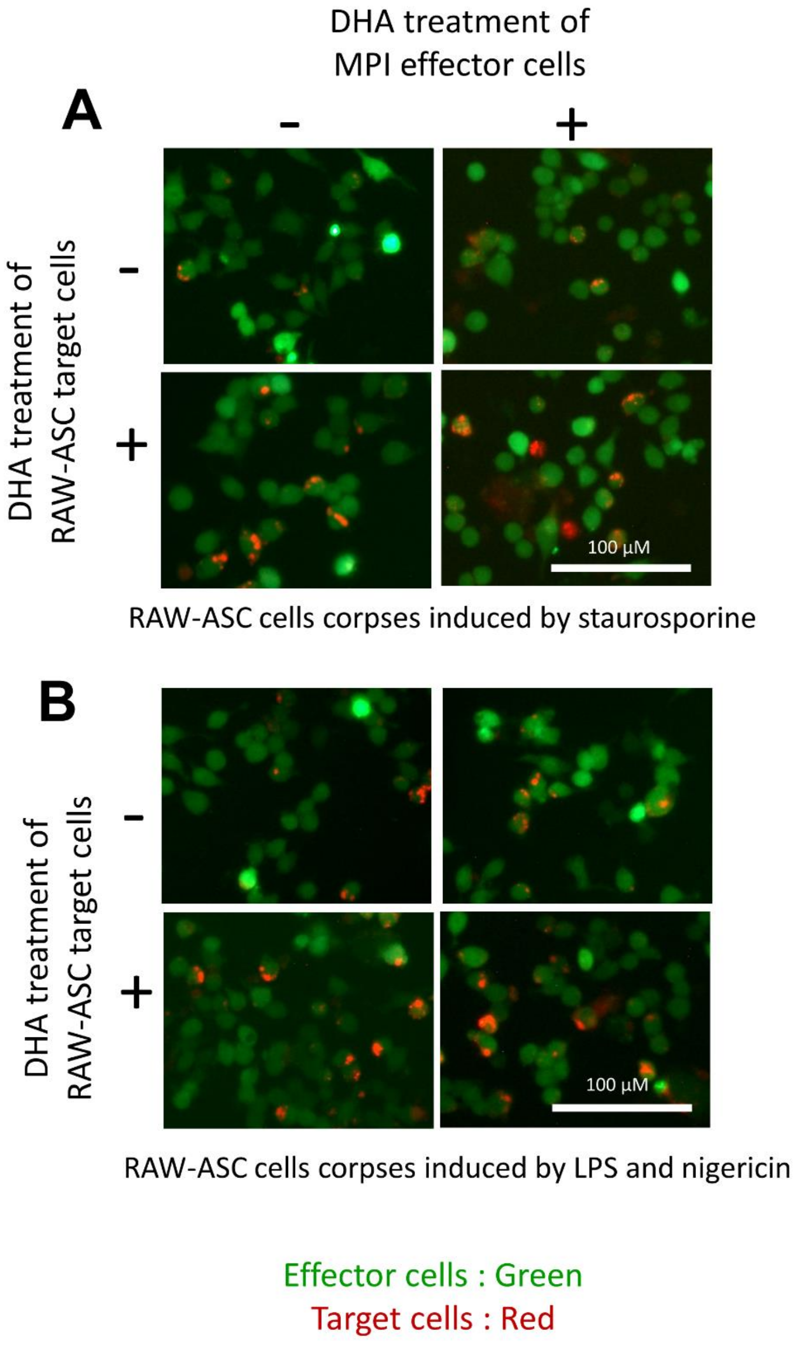

Supplemental Fig 7

Supplementary Figure 7: DHA enhances efferocytotic activity of MPI effector cells when RAW-ASC target cells are pre-incubated with DHA. Effect of DHA pretreatments on MPI cell engulfment of apoptotic RAW-ASC cells induced by (A) staurosporine and **(B)** LPS+ nigericin. Merged photomicrograph images were acquired using the EVOS FL 2 microscope (20X) showing pHrodo Red SE-labeled target cells (red) engulfed by the MPI cells (green). Images were taken at 20x magnification with a representative portion shown. Similar results were obtained in two independent experiments.
